# STING Mediates Lupus via the Activation of Conventional Dendritic Cell Maturation and Plasmacytoid Dendritic Cell Differentiation

**DOI:** 10.1016/j.isci.2020.101530

**Published:** 2020-09-04

**Authors:** Arthid Thim-uam, Thaneas Prabakaran, Mookmanee Tansakul, Jiradej Makjaroen, Piriya Wongkongkathep, Naphat Chantaravisoot, Thammakorn Saethang, Asada Leelahavanichkul, Thitima Benjachat, Søren Paludan, Trairak Pisitkun, Prapaporn Pisitkun

**Affiliations:** 1 Interdisciplinary Program of Biomedical Sciences, Graduate School, Chulalongkorn University, 1873 Rama 4 Road, Pathumwan, Bangkok 10330, Thailand; 2 Center of Excellence in Systems Biology, Faculty of Medicine, Chulalongkorn University, 1873 Rama 4 Road, Pathumwan, Bangkok 10330, Thailand; 3 Department of Biomedicine, Aarhus University, Aarhus 8000, Denmark; 4 Section for Translational Medicine Program, Faculty of Medicine Ramathibodi Hospital, Mahidol University, 270 Rama 6 Road, Ratchathewi, Bangkok 10400, Thailand; 5 Department of Biochemistry, Faculty of Medicine, Chulalongkorn University, 1873 Rama 4 Road, Pathumwan, Bangkok 10330, Thailand; 6 Center of Excellence in Immunology and Immune-mediated Diseases, Faculty of Medicine, Chulalongkorn University, 1873 Rama 4 Road, Pathumwan, Bangkok 10330, Thailand; 7 Epithelial Systems Biology Laboratory, National Heart, Lung and Blood Institute, National Institutes of Health, Bethesda, MD, USA; 8 Division of Allergy, Immunology, and Rheumatology, Department of Medicine, Faculty of Medicine Ramathibodi Hospital, Mahidol University, 270 Rama 6 Road, Ratchathewi, Bangkok 10400, Thailand

**Keywords:** Immunology, Molecular Genetics, Molecular Biology

## Abstract

Signaling through stimulator of interferon genes (STING) leads to the production of type I interferons (IFN-Is) and inflammatory cytokines. A gain-of-function mutation in STING was identified in an autoinflammatory disease (STING-associated vasculopathy with onset in infancy; SAVI). The expression of cyclic GMP-AMP, DNA-activated cGAS-STING pathway, increased in a proportion of patients with SLE. The STING signaling pathway may be a candidate for targeted therapy in SLE. Here, we demonstrated that disruption of STING signaling ameliorated lupus development in *Fcgr2b*-deficient mice. Activation of STING promoted maturation of conventional dendritic cells and differentiation of plasmacytoid dendritic cells via LYN interaction and phosphorylation. The inhibition of LYN decreased the differentiation of STING-activated dendritic cells. Adoptive transfer of STING-activated bone marrow-derived dendritic cells into the FCGR2B and STING double-deficiency mice restored lupus phenotypes. These findings provide evidence that the inhibition of STING signaling may be a candidate targeted treatment for a subset of patients with SLE.

## Highlights

• STING constitutively activates in the Fcgr2b-deficient lupus mice
• Signaling through STING-LYN interaction promotes DC differentiation
• Inhibition of STING pathway disrupts the lupus phenotypes
• STING mediates lupus disease via the activation of dendritic cells

## Graphical Abstract

**Figure:**
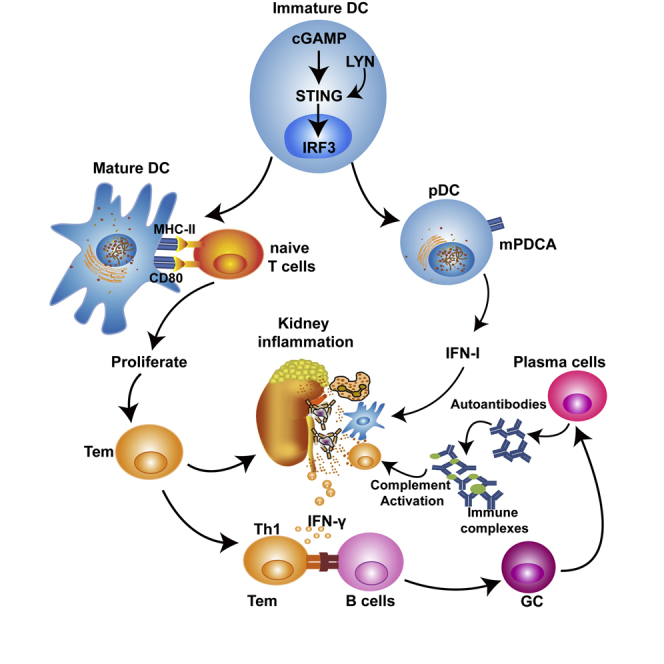

## Introduction

Systemic lupus erythematosus (SLE) is an autoimmune disease with characteristics of autoantibody production and immune complex deposition that lead to severe inflammation and fatal glomerulonephritis. The heterogeneity of lupus disease has been shown through several mouse models of lupus disease, suggesting a variety of unique mechanisms participating in its pathogenesis. Type I interferon (IFN-I) is known to play significant roles in SLE pathogenesis (Muskardin and Niewold, 2018). In many patients with SLE, the expression of interferon-inducible genes is increased in their peripheral blood mononuclear cells (Baechler et al., 2003). Nucleic acid-sensing pathways are the main contributors of IFN-I production (Paludan, 2015). Several studies suggest that inappropriate recognition of self-nucleic acids can induce the production of IFN-I and promote SLE disease (Muskardin and Niewold, 2018).

Nucleic acids derived from extracellular sources are sensed via endosomal Toll-like-receptors (TLRs), whereas the recognition of cytosolic nucleic acids is independent of TLRs (Keating et al., 2011). The activation of TLRs, such as TLR7 and TLR9, by endosomal nucleic acids, leads to type I interferon production (Asselin-Paturel et al., 2005). Spontaneous duplication of *Tlr7* causes autoimmune lupus phenotypes in *Yaa*-carrying BXSB mice (Pisitkun et al., 2006). Overexpression of *Tlr7* promotes autoimmunity through dendritic cell proliferation, whereas the deletion of *Tlr7* in lupus-prone MRL/*lpr* mice diminishes autoantibody and immune activation (Christensen et al., 2006; Deane et al., 2007). However, blocking TLR-mediated signaling by anti-malarial drugs can only treat SLE with mild disease activity (Sacre et al., 2012; Zeidi et al., 2019). Thus, investigation of other nucleic acid sensor pathways involved in lupus development could offer a more significant therapeutic opportunity.

Several cytosolic DNA sensors can induce IFN-I production, with cyclic GMP-AMP synthase (cGAS) being the major one (Sun et al., 2013). Cytosolic DNA sensing is also essential for innate immune signaling, and dysregulation of this process can cause autoimmune and inflammatory diseases (Yan, 2017). Stimulator of interferon genes (STING), also known as transmembrane protein 173 (TMEM173), is a cytoplasmic adaptor protein that acts downstream of cGAS to enhance IFN-I production (Ishikawa et al., 2009). The loss-of-function mutations in a DNA-specific exonuclease gene *TREX1*, resulting in increased cytosolic DNA levels, are observed in the type I interferonopathies Aicardi-Goutieres syndrome (AGS) and chilblain lupus (Crow et al., 2006; Günther et al., 2013). Consistent with these scenarios in human, *Trex1*-deficient mice exhibit fatal inflammation and autoimmunity (Morita et al., 2004; Stetson et al., 2008). Inhibition of the STING pathway in these mice improves their inflammatory condition and survival (Ahn et al., 2014). Moreover, the absence of STING rescues embryonic lethality and arthritis development in another nuclease knockout model, i.e., *DNase II*-deficient mice (Ahn et al., 2012).

The spontaneous lupus mouse models commonly used to study SLE pathogenesis are MRL/*lpr*, NZBxNZW.F1, and BxSB (Theofilopoulos and Dixon, 1985). Since these models possess different genetic backgrounds, each model could develop lupus with unique pathogenesis (Murphy and Roths, 1979; Pisitkun et al., 2006; Theofilopoulos and Dixon, 1985). Surprisingly, the absence of STING in MRL/*lpr* mice does not improve lupus phenotypes but instead promotes more inflammation (Sharma et al., 2015). Furthermore, knocking down the IFN receptor gene *Ifnar1* in MRL/*lpr* mice aggravates lymphoproliferation, autoantibody production, and end-organ damage (Hron and Peng, 2004; Nickerson et al., 2010). Although the expression of cyclic GMP-AMP, DNA-activated cGAS-STING pathway, activated IFN-I, and increased in a proportion of patients with SLE (An et al., 2017), the data from lupus mouse models reveal the differential roles of STING in lupus pathogenesis depending on the models. Therefore, further studies in a relevant animal model that reflects human lupus are required to circumvent these conflicting data.

A comprehensive genetic analysis has identified *FCGR2B* as a susceptibility gene in patients with SLE (Zhu et al., 2016). The deletion of the *Fcgr2b* gene causes a lupus-like disease in genetic susceptibility to autoimmune development. The *Fcgr2b*^*−/−*^ mice created in 129 strain with subsequently backcrossed into C57BL/6 develop overt autoreactivity and fatal lupus disease while the deletion of *Fcgr2b* in C57BL/6 mice showed only autoantibody production (Bolland and Ravetch, 2000; Boross et al., 2011). The 129-derived Sle16 covering the Nba2 interval region is an autoimmune susceptibility locus, which contains the *Fcgr2b*, Slam family, interferon-inducible Ifi200 family genes (Boross et al., 2011; Choubey, 2012; Sato-Hayashizaki et al., 2011). Among the Ifi200 family, the *Ifi202* shows the highest expression in the splenocytes from Nba2 carrying mice (Rozzo et al., 2001). The *Ifi202* is a candidate lupus susceptibility gene, and its human homolog *IFI16* shows the association with SLE (Kimkong et al., 2010). Also, IFI16 signals through STING to initiate IFN-I production (Choubey and Panchanathan, 2008; Unterholzner et al., 2010). Based on the genetic background of the 129-derived locus, STING may play a significant role in the pathogenesis of the 129/B6.*Fcgr2b*^*−/−*^ lupus mice.

In this work, we showed an increase in *Sting* expression of the 129/B6.*Fcgr2b*^*−/−*^ mice. Disruption of STING signaling rescued lupus phenotypes of the 129/B6.*Fcgr2b*^*−/−*^ mice. Stimulation of STING promoted dendritic cell maturation and plasmacytoid dendritic cell differentiation. After STING activation, LYN was phosphorylated and recruited to interact with STING. Inhibition of LYN diminished STING-driven differentiation of dendritic cells. The adoptive transfer of STING-activated bone marrow-derived dendritic cells (BMDCs) into the double-deficiency (*Fcgr2b*^*−/−*^*.Sting*^*gt/gt*^) mice restored the lupus phenotypes. The data suggested that STING signaling in the dendritic cells initiated the autoimmune development in the 129/B6.*Fcgr2b*^*−/−*^ mice. STING is a promising therapeutic target for lupus disease.

## Results

### Loss of the Stimulator of Type I Interferon Genes (STING) Increases Survival of *Fcgr2b*^*−/−*^ Lupus Mice

**Figure 1:**
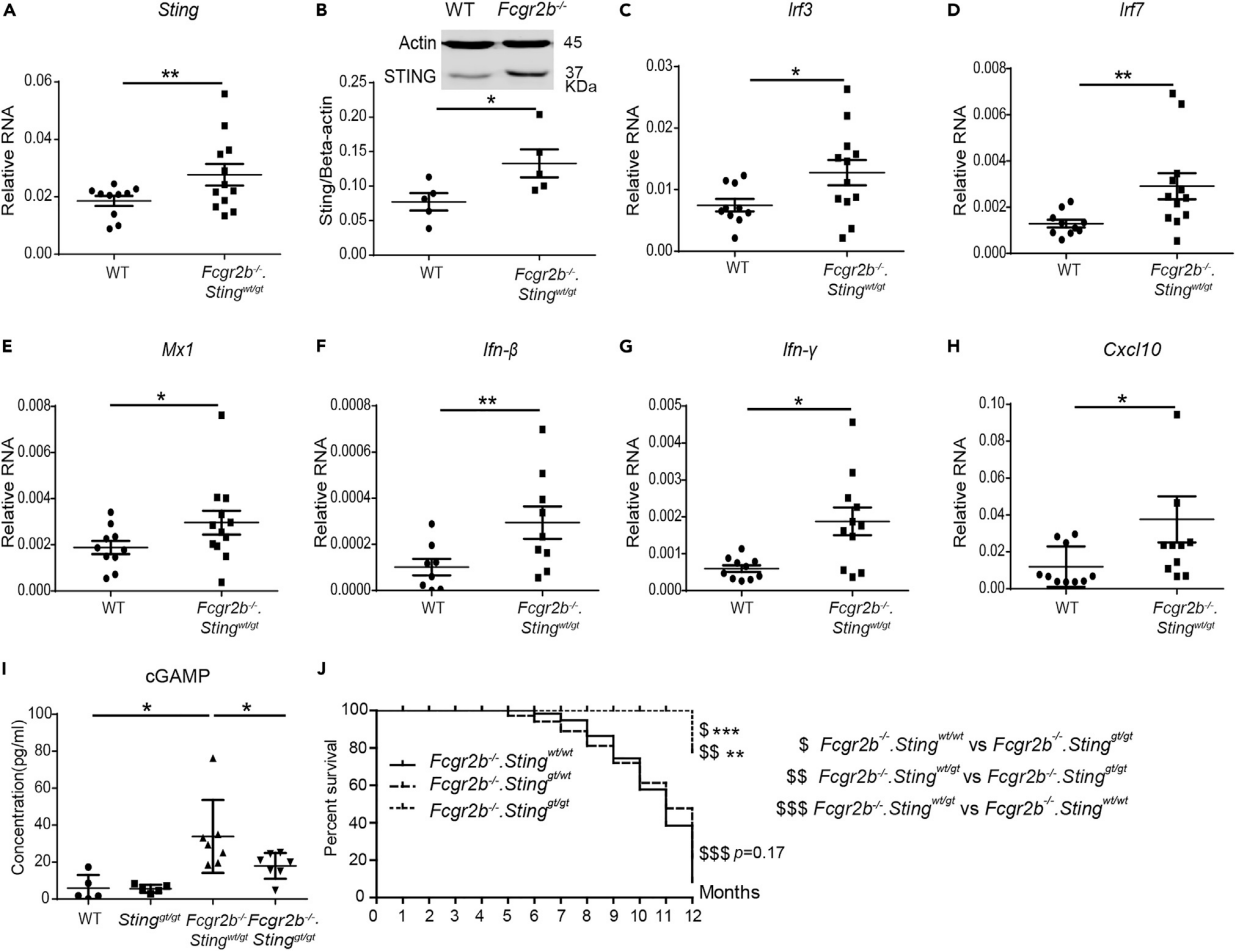
Loss of the Stimulator of Type I Interferon Genes (STING) Increases Survival of *Fcgr2b*^*−/−*^ Lupus Mice (A and C–H) Gene expression profiles from spleens of wild-type and *Fcgr2b*^*−/−*^ mice at the age of 6 months were tested by real-time PCR (N = 10–12 per group). The relative RNA expressions (normalized by actin) of (A) *Sting*, (C) *Irf3*, (D) *Irf7*, (E) *Mx1*, (F) *Ifn-β*, (G) *Ifn-γ*, and (H) *Cxcl10* are shown. (B) Isolated splenocytes were analyzed for STING protein expression by western blot. Data are representative of three mice per group. Quantification of the intensity was normalized by actin (N = 3 per group). (I) The concentration of cGAMP from isolated splenocytes (N = 5–7). (J) The *Fcgr2b*-deficient mice were crossed with *Sting*-deficient mice (*Sting*^*gt/gt*^) to generate the double-deficient mice (*Fcgr2b*^*−/−*^*. Sting*^*gt/gt*^) and littermate controls. The survival curve of the mice was observed for up to 12 months (N = 14 per group). Error bars indicate SEM; ∗p < 0.05, ∗∗p < 0.01, and ∗∗∗p < 0.001. The dollar sign ($) shown the comparison between the group.

First, we confirmed that 129/B6.*Fcgr2b*^*−/−*^ mice (or *Fcgr2b*^*−/−*^ in short) showed the increase of *Sting mRNA expression and protein expression in the spleen* (Figures 1A and 1B). We further observed the significant rise of mRNA expression of interferon-inducible genes (*Irf3*, *Irf7, Mx1*) (*Ifn-γ, Ifn-β,* and *Cxcl10*) and in the spleen of the *Fcgr2b*^*−/−*^ mice (Figures 1C–1H). To determine whether the Sting signaling is required for lupus development in the *Fcgr2b*^*−/−*^ mice, we generated the double deficiency of *Fcgr2b* and *Sting* together with control littermates. The *Fcgr2b*^*−/−*^ mice were crossed with the C57BL/6.*Sting* deficiency or Goldenticket mice (*Sting*^*gt*^), which behave as a functional knockout of STING (Sauer et al., 2011). Furthermore, we detected the increase of cGAMP in the splenocytes of the *Fcgr2b*^*−/−*^*.Sting*^*wt/gt*^ mice but not in the double-deficient mice (Figure 1I). The double-deficient mice (*Fcgr2b*^*−/−*^*.Sting*^*gt/gt*^) showed a higher survival rate compared with the *Fcgr2b*^*−/−*^ with homozygote *Sting* WT mice (Figure 1J). Also, the *Fcgr2b*^*−/−*^ with heterozygote of Sting (*Sting*^*wt/gt*^) showed similarity in phenotypes and survival with *Fcgr2b*^*−/−*^ with wild-type Sting (*Sting*^*wt/wt*^).

### STING Signaling Pathway Promotes Autoantibody Production and Glomerulonephritis in the *Fcgr2b*^*−/−*^ Lupus Mice

**Figure 2:**
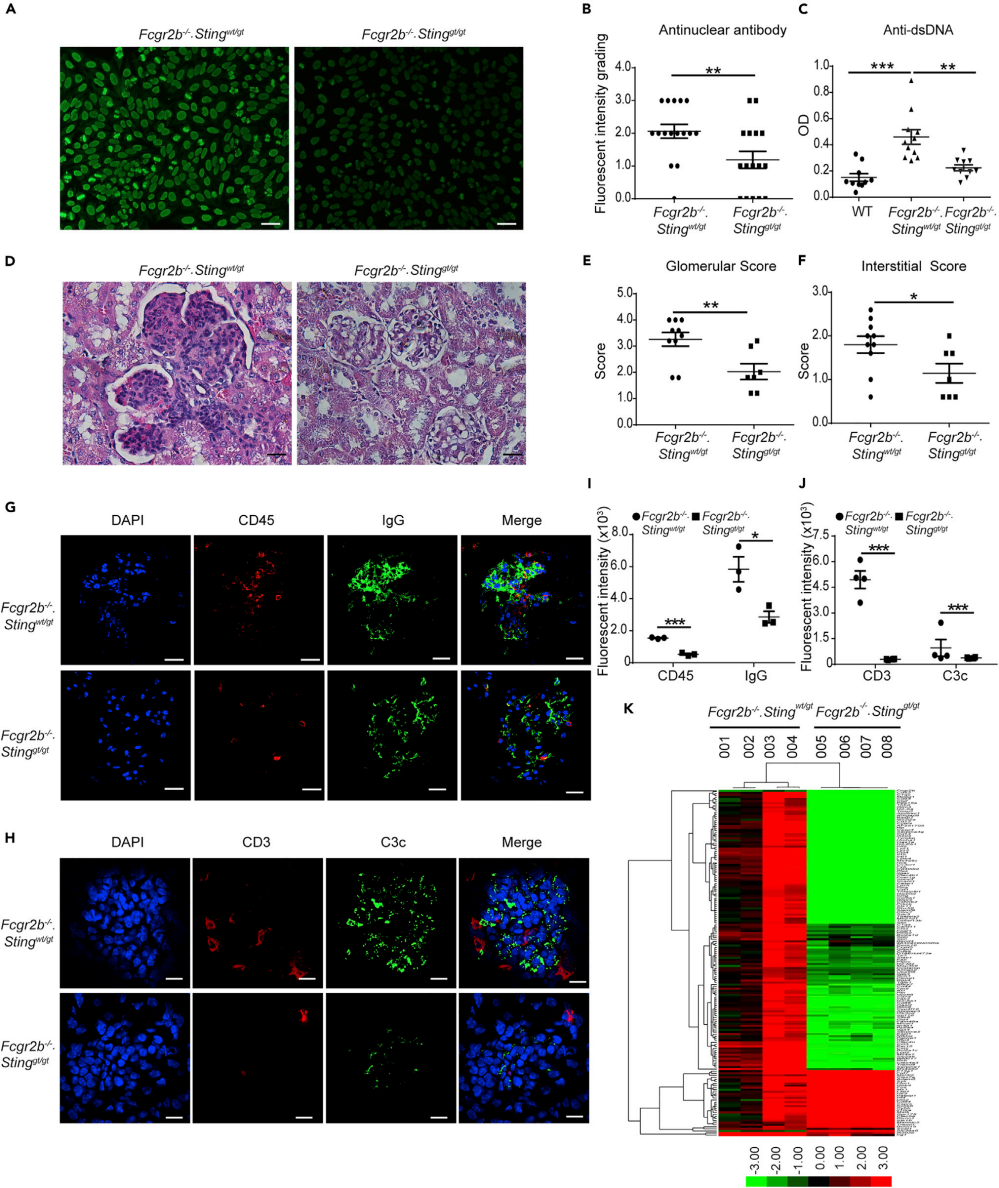
STING Signaling Pathway Promotes Autoantibody Production and Glomerulonephritis in the *Fcgr2b*^*−/−*^ Lupus Mice (A)The anti-nuclear antibodies (ANA) were detected in the serum (dilution 1:800) using the immunofluorescence staining on Hep-2 cells (A). Data are representative of eight mice per group (scale bar, 20 μm). (B) Semi-quantification of ANA was graded by fluorescence intensity (N = 8 mice per group). (C) Anti-dsDNA from sera (dilution 1:100) of *Fcgr2b*^*−/−*^ and *Fcgr2b*^*−/−*^*. Sting*^*gt/gt*^ was detected by ELISA (N = 10–11 per group). (D) Kidney sections of *Fcgr2b*^*−/−*^ and *Fcgr2b*^*−/−*^*. Sting*^*gt/gt*^ mice (6–8 months old) were stained with H&E. Data are representative of 7–10 mice per group (scale bar, 25 μm). (E–H) (E and F) Glomerular scores and interstitial scores of kidney sections were blindly graded (N = 7–10 per group). Immunofluorescence staining of the kidneys from *Fcgr2b*^*−/−*^ and *Fcgr2b*^*−/−*^*. Sting*^*gt/gt*^ mice show in (G) IgG (green), CD45 (red), and DAPI (blue) and (H) C3c (green), CD3 (red), and DAPI (blue). Data are representative of 3–4 mice per group (scale bar, 10 μm). (I and J) The quantitative immunofluorescence signal (I) CD45, and IgG, (J) CD3 and C3c (N = 3–4 mice per group). Data are shown as mean ± SEM; ∗p < 0.05, ∗∗p < 0.01 and ∗∗∗p < 0.001. (K) A heatmap of microarray data from the kidneys of *Fcgr2b*^*−/−*^ and *Fcgr2b*^*−/−*^*. Sting*^*gt/gt*^ mice show that the interferon signature genes significantly changed in the *Fcgr2b*^*−/−*^mice (N = 4 mice per group). Data shown in log_2_ (sample/wild-type).

The lupus phenotypes of the double-deficient mice were examined and compared with littermate controls. The levels of the anti-nuclear antibody (ANA) and anti-dsDNA antibody from the sera of the double-deficient mice (*Fcgr2b*^*−/−*^*.Sting*^*gt/gt*^) were significantly lower than in the *Fcgr2b*^−/−^ mice (Figures 2A–2C). The kidneys of *Fcgr2b*^*−/−*^ mice showed pathology of diffuse proliferative glomerulonephritis, which did not present in the *Fcgr2b*^*−/−*^.*Sting*^*gt/gt*^ mice (Figure 2D). The glomerular and interstitial scores in the kidneys of *Fcgr2b*^*−/−*^ mice were significantly higher than in the double-deficient mice (Figures 2E and 2F). Consistent with the pathology, the immunofluorescence staining showed fewer CD45^+^ cells and IgG deposition in the kidneys of *Fcgr2b*^*−/−*^.*Sting*^*gt/gt*^ mice (Figures 2G and 2I). The cell types infiltrated in the kidneys of the *Fcgr2b*^*−/−*^ mice were CD3^+^ and CD11c^+^ cells, which significantly reduced in the double-deficient mice (Figures 2H, 2J, S1B, and S1C. Related to Figure 2).

We further looked at the gene expression profiles in the kidney of these mice and found a significantly different expression (Figure S1A. Related to Figure 2). The expression of interferon-inducible genes in the kidney of these mice was higher in the *Fcgr2b*^*−/−*^ mice, especially in the ones with greater severity (#003, 004), and there was a significant reduction of interferon-inducible genes in the kidneys of *Fcgr2b*^*−/−*^.*Sting*^*gt/gt*^ mice (Figure 2K). However, not all of these interferon-inducible genes decreased in the *Fcgr2b*^*−/−*^.*Sting*^*gt/gt*^ mice (Figure 2K). These data suggested that STING-dependent pathology mediated by both interferon and non-interferon signaling and not all of the interferon signaling in the *Fcgr2b*^*−/−*^ mice contributed by the STING pathway.

### STING Is Essential for Inflammatory Phenotypes of the *Fcgr2b*^*−/−*^ Lupus Mice

**Figure 3:**
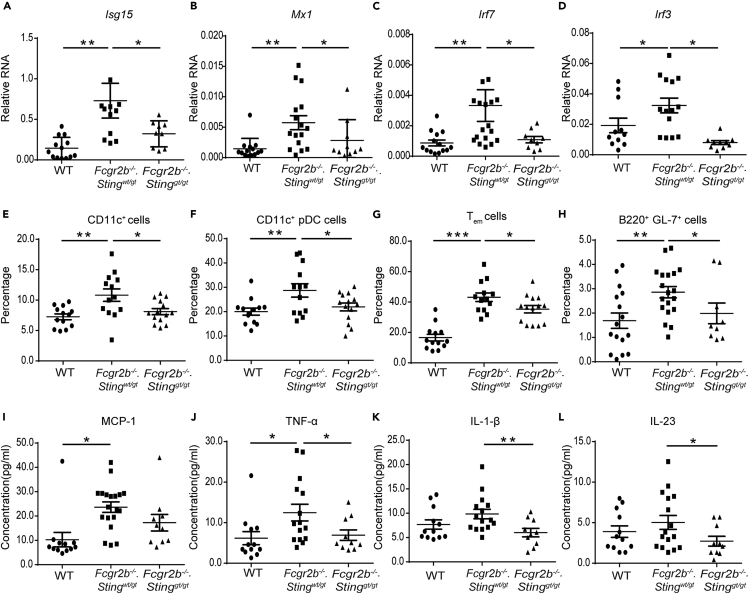
STING is Essential for Inflammatory Phenotypes of the *Fcgr2b*^*−/−*^ Lupus Mice (A–D) The relative RNA expression (normalized by actin) of (A) *Isg15*, (B) *Mx1*, (C) *Irf7*, and (D) *Irf3* from the kidneys of wild-type, *Fcgr2b*^*−/−*^*. Sting*^*wt/gt*^, and *Fcgr2b*^*−/−*^*. Sting*^*gt/gt*^ mice at the age of 6 months are shown (N = 10–17 per group). (E–H) Flow cytometry analysis of splenocytes isolated from wild-type, *Fcgr2b*^*−/−*^*. Sting*^*wt/gt*^, and *Fcgr2b*^*−/−*^*. Sting*^*gt/gt*^ mice at the age of 6–7 months (N = 13–14 per group). Data are shown in the percentage of (E) CD11c^+^, (F) plasmacytoid dendritic cells (pDC), (G) T_em_ (CD3^+^CD4^+^CD44^hi^CD62L^lo^), and (H) B220^+^GL7^+^ cells. (I–L) The sera cytokines of wild-type, *Fcgr2b*^*−/−*^*. Sting*^*wt/gt*^, and *Fcgr2b*^*−/−*^*. Sting*^*gt/gt*^ mice at the age of 6 months were analyzed by cytometric bead array. Serum cytokines of (I) MCP-1, (J) TNF-α, (K) IL-1β, and (L) IL-23 (N = 10–15 per group). Data are shown as mean ± SEM; ∗p < 0.05, ∗∗p < 0.01 and ∗∗∗p < 0.001.

The expression of interferon-inducible genes and interferon regulatory factors in the kidneys was confirmed by real-time PCR. The expressions of *Isg15*, *Mx1, Irf7,* and *Irf3* were upregulated in the *Fcgr2b*^*−/−*^ mice and downregulated in the absence of STING (Figures 3A–3D). Also, the expression of *Irf5*, the lupus susceptibility gene, which upregulated in the kidneys of the *Fcgr2b*^*−/−*^ mice, was Sting dependent (Figure S2A. Related to Figure 3). The splenocytes were analyzed from the mice at the age of 6–7 months to characterize the immunophenotypes. The expansion of dendritic cells (CD11c^+^) and plasmacytoid dendritic cells (CD11c^+^PDCA^+^) in the *Fcgr2b*^*−/−*^ mice diminished in the absence of Sting (Figures 3E and 3F). The reduction of T effector memory cells (CD3^+^CD4^+^CD62L^lo^CD44^hi^), germinal center B cells (B220^+^GL7^+^), and CD4^+^ICOS^+^ cells in the double-deficient mice were detected (Figures 3G, 3H, and S2B. Related to Figure 3). The percentage of IAb^+^ B cells significantly reduced in the double-deficient mice (Figure S2C. Related to Figure 3). However, the expansion of plasma cells did not show the difference between single and double-deficient mice (Figure S2D. Related to Figure 3). Besides, the percentage of CD11b^+^CD11c^−^ and F480^+^ cells did not increase in both *Fcgr2b*^*−/−*^*.Sting*^*wt/gt*^ mice and double-deficient mice (Figures S2E and S2F. Related to Figure 3). Furthermore, the sera levels of MCP-1 and TNF-α from the *Fcgr2b*^*−/−*^*.Sting*^*wt/gt*^ mice were significantly increased compared with WT mice (Figures 3I and 3J), whereas IL-1β and IL-23 did not show significant changes (Figures 3K and 3L). However, the levels of TNF-α, IL-1β, and IL-23 from the *Fcgr2b*^*−/−*^ mice significantly decreased in the absence of STING (Figures 3J–3L). These data suggested that STING mediated the inflammatory process in the *Fcgr2b*-deficient lupus mice.

### STING-Activated Dendritic Cells Induce the Proliferation of Naive CD4^+^ T Cells

**Figure 4:**
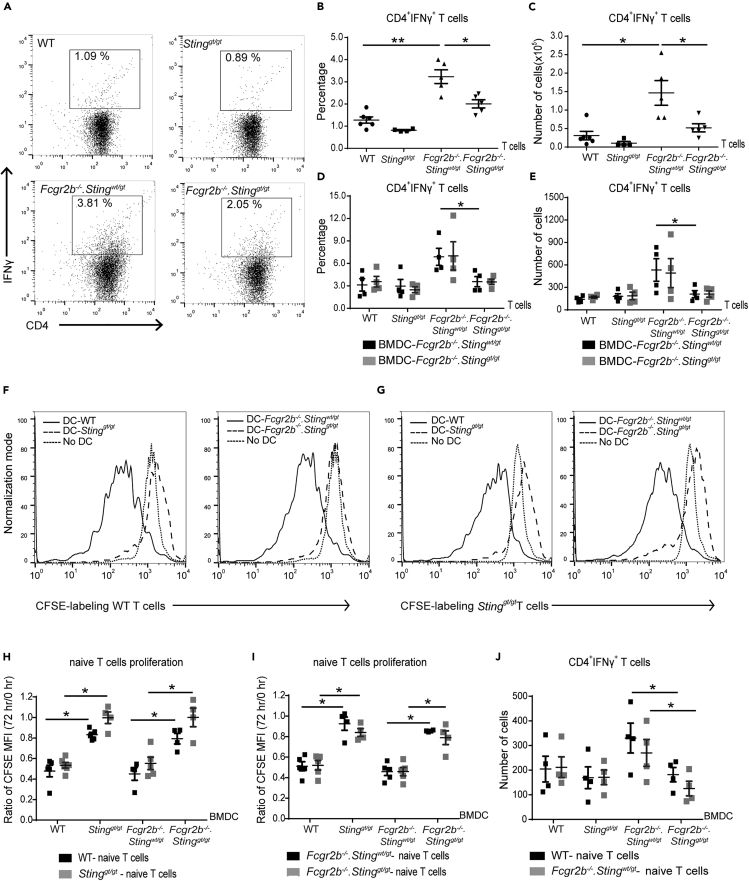
STING-Activated Dendritic Cells Induce the Proliferation of Naive CD4^+^ T Cells (A–C) Flow cytometry analysis of (A and B) intracellular staining of IFN-γ-producing CD4^+^ T cells isolated from lymph nodes of wild-type, *Sting*^*gt/gt*^, *Fcgr2b*^*−/−*^*.Sting*^*wt/gt*^, and *Fcgr2b*^*−/−*^*.Sting*^*gt/gt*^ mice at the age of 6–7 months. (A) Data are representative of 4–5 mice per group. (B) The percentage of IFN-γ^+^CD4^+^ T cells and (C) the number of IFN-γ^+^CD4^+^ T cells (N = 4–5 per group). (D and E) The isolated CD4^+^ T cells were co-cultured with stimulated BMDC for 6 h. The x axis shows the genotypes that CD4^+^ T cells were isolated. (D) The percentage and (E) the number of intracellular IFN-γ-producing CD4^+^ cells after co-culturing with DMXAA-activated BMDC from *Fcgr2b*^*−/−*^*.Sting*^*wt/gt*^ and *Fcgr2b*^*−/−*^*.Sting*^*gt/gt*^ (6–7 months old) for 6 h (N = 4–5). (F–J) Co-culture of naive T cells with DMXAA-activated BMDC from wild-type, *Sting*^*gt/gt*^, *Fcgr2b*^*−/−*^*.Sting*^*wt/gt*^, and *Fcgr2b*^*−/−*^*.Sting*^*gt/gt*^ mice for 72 h. The x axis shows the genotypes that BMDCs were isolated. (F and G) The histogram of CFSE labeling T cells in the co-culture with BMDCs. Data are representative of 4–5 mice per group. (H and I) CFSE dilution of isolated naive T cells showed in the ratio of mean fluorescence intensity (MFI) at 72 h/initial labeling (time 0), and (J) the total numbers of IFN-γ^+^CD4^+^ T cells (N = 4 per group). Data are shown as mean ± SEM; ∗p < 0.05, and ∗∗p < 0.01.

The splenocytes of Fcgr2b−/− mice showed an increase of effector memory T cells (Tem) and Ifng expression (Figures 3G and 1G). We hypothesized that the high proportion of Tem in the Fcgr2b−/− mice might contribute to the increase of Ifng expression. We performed the intercellular staining of IFN-γ to test this assumption. The IFN-γ^+^CD4^+^ T cells from the lymph nodes of Fcgr2b−/− mice were higher than wild-type and double-deficient mice (Figures 4A–4C). These IFN-γ^+^CD4^+^ T cells isolated from lymph nodes were primed *in vivo* by DCs. The reduction of IFN-γ^+^CD4^+^ T cells in the double-deficient mice could suggest that either STING in T cells or DCs could play a role in the *in vivo* Th1 skewing.

Sting deficiency reduced the DC expansion in the spleen of Fcgr2b−/− mice (Figure 3E). We hypothesized that DC of the Fcgr2b−/− mice might promote the expansion of Tem and IFN-γ^+^ T cells. Thus, we co-cultured T cells with BMDC to check if the STING-expressing DC could directly influence the IFN-γ production in T cells. The co-culture for 6 h between CD4^+^ T cells (from every genotype) and STING-activated BMDC from either Fcgr2b−/− or double-deficient mice showed similar numbers of IFN-γ^+^CD4^+^ T cells (Figures 4D and 4E). However, the number of IFN-γ^+^CD4^+^ T cells isolated from Fcgr2b−/− mice in the co-culture with BMDC was higher than the isolated CD4^+^ cells from double-knockout mice regardless of STING expression on BMDC (Figures 4D and 4E). These data suggested that T cell priming of whole CD4^+^ T cells by DCs was not affected by the presence or absence of STING because Th1 cells in *Fcgr2b*^*−/−*^ T cells were fully differentiated *in vivo*. This experiment suggested that STING-expressing DC could not promote the primed T cells *in vivo* to produce more IFN-γ.

Next, we tested if STING-expressing DC could prime naive T cells to proliferate and differentiate into IFN-γ-producing T cells. The purified naive T cells were labeled with CFSE and co-cultured with STING-activated BMDC for 72 h. The CFSE dilution assay showed that STING-expressing DC induced the proliferation of naive T cells regardless of STING expression on T cells (Figures 4F–4I). Interestingly, only BMDC from the Fcgr2b−/− mice induced the differentiation of Th1 cells regardless of the STING expression on naive T cells (Figure 4J). These co-culture experiments indicated that intrinsic STING expression on DC, but not on T cells, promoted Th1 differentiation. The data suggested that STING activation promoted the maturation of DC in the Fcgr2b−/− mice, which subsequently primed the naive CD4^+^ T cells to proliferate and become the IFN-γ-producing T cells.

### STING Activation Promotes the Maturation of Dendritic Cells and the Differentiation of Plasmacytoid Dendritic Cells

**Figure 5:**
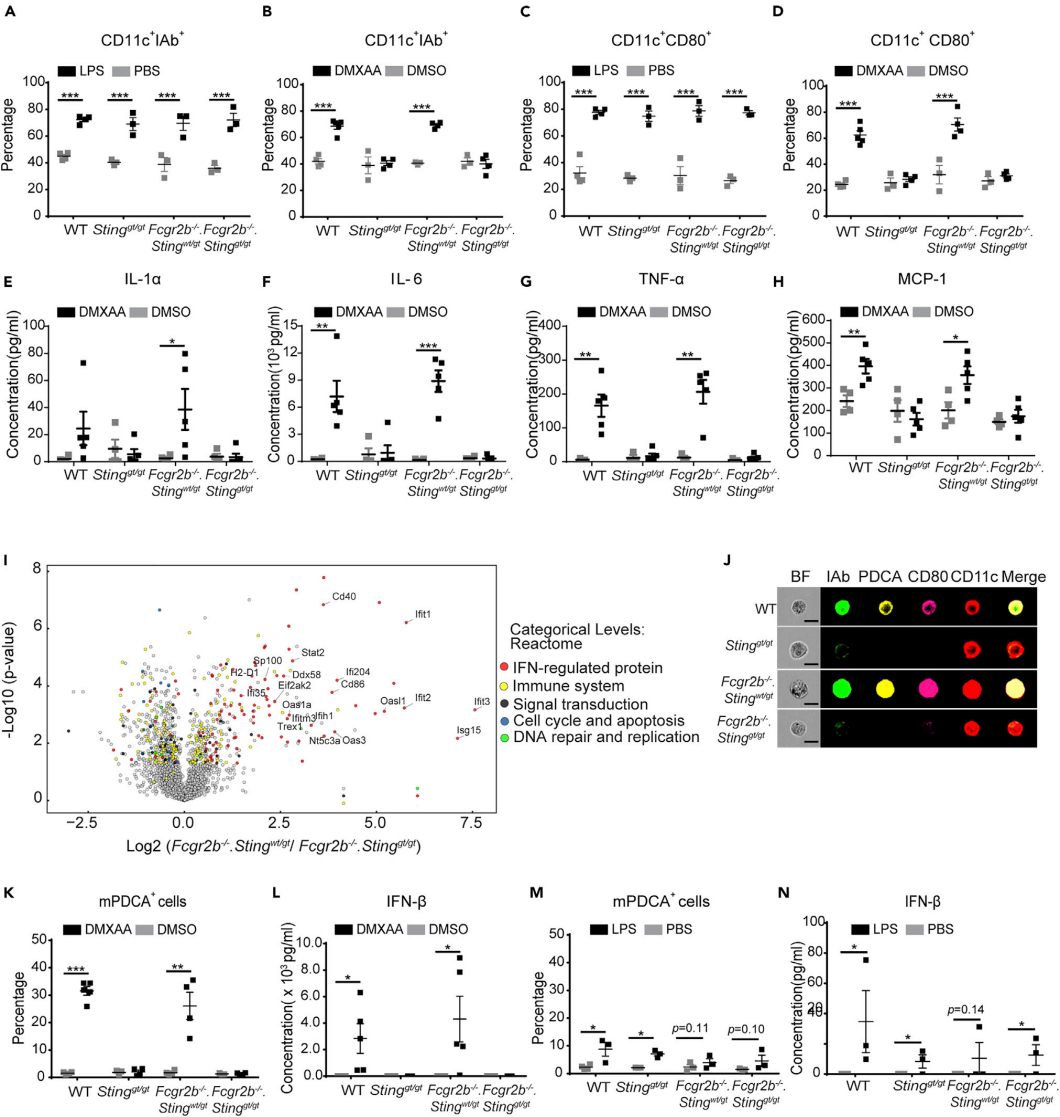
STING Activation Promotes the Maturation of Dendritic Cells and the Differentiation of Plasmacytoid Dendritic Cells Bone marrows were isolated from wild-type, *Sting*^*gt/gt*^, *Fcgr2b*^*−/−*^*. Sting*^*wt/gt*^, and *Fcgr2b*^*−/−*^*. Sting*^*gt/gt*^ mice at the age of 6 months. (A–D) IL-4 and G-CSF differentiated bone marrow-derived dendritic cells (BMDC) for 5 days were stimulated with LPS or DMXAA for 24 h. Flow cytometry analysis shows the percentage of (A and B) CD11c^+^ IAb^+^ cells and (C and D) CD11c^+^CD80^+^ cells. (E–H) Supernatants were collected and analyzed after DMXAA stimulation for 24 h. Cytometric bead array shows the levels of (E) IL-1α, (F) IL-6, (G) TNF-α, and (H) MCP-1. (I) Volcano plot of protein expressions from proteomic analysis of DMXAA-activated BMDC of *Fcgr2b*^*−/−*^*. Sting*^*wt/gt*^, and *Fcgr2b*^*−/−*^.*Sting*^*gt/gt*^ mice at the age of 6–7 months (N = 4 per group). (J) Imaging flow cytometry of DMXAA-activated BMDC shows the representative staining of IAb (green), mPDCA (yellow), CD80 (pink), and CD11c (red) (N = 3 mice per group). (K and M) The percentage of pDC (PDCA^+^ cells) after (K) DMXAA activation and (M) LPS activation for 24 h (N = 3–4 per group). (L and N) The level of IFN-β from the culture supernatant of activated BMDC with (L) DMXAA and (N) LPS (N = 5 per group). Data are shown as mean ± SEM; ∗p < 0.05, ∗∗p < 0.01 and ∗∗∗p < 0.001.

The cGAS/STING pathway is essential for DC activation (Marinho et al., 2018). STING-activating DC can induce naive T cells to proliferate and produce IFN-γ (Figures 4H–4J), which suggested STING may enhance the maturation of DC to become professional antigen-presenting cells. We hypothesized that STING signaling could mediate the expansion of DC in the *Fcgr2b*^*−/−*^ mice. The bone-marrow-derived dendritic cells (BMDCs) were differentiated into immature DCs and subsequently stimulated with STING ligands (DMXAA), DMSO, and LPS (as a control) to assess if STING played a role in DC maturation. The LPS control induced the immature DC to increase the expression of MHC-II (IA-b) and CD80, which suggested the phenotypes of mature DC, from both *Sting*-sufficient and *Sting*-deficient mice (Figures 5A and 5C). The immature DC from wild-type and *Fcgr2b*^*−/−*^ mice also showed the increasing percentage of IA-b^+^ and CD80^+^ DC cells after DMXAA stimulation; the *Sting*-deficient mice did not develop these mature phenotypes (Figures 5B and 5D). The supernatant from BMDC culture with DMXAA stimulation showed an increase in the concentration of IL-1α, IL-6, TNF-α, and MCP-1 in the wild-type and *Fcgr2b*^*−/−*^ mice but not in *Sting*-deficient mice and double-deficient mice (Figures 5E–5H).

To better understand the mechanisms of STING in DC differentiation, we performed the proteomic analysis of STING-activated BMDC in the *Fcgr2b*^*−/−*^ mice compared with the double-deficient mice. The volcano plot showed the proteins that were highly expressed were interferon-regulated proteins (Figure 5I and Table S1. Lists of up and down of regulated proteins. Related to Figure 5). This finding may result from the increase of IFN-I production in the culture medium, which could upregulate the interferon-regulated proteins. We hypothesized that STING might promote the differentiation of pDC (a significant producer of IFN-I). The *in vitro* culture of BMDC with DMXAA and LPS (as a control) showed a significant increase in pDC and IFN-β with DMXAA but not with LPS stimulation (Figures 5K–5N). Also, we demonstrated the morphology of these cells by the imaging flow cytometry and found the pDCs expressed CD80 as well (Figure 5J). To confirm that activation of STING via another ligand results in DC differentiation, we stimulated the BMDC with cGAMP. The stimulation of STING with cGAMP increased the amount of CD11c^+^IAb^+^ (Figure S3A. Related to Figure 5), CD11c^+^CD80^+^ (Figure S2B. Related to Figure 5), and mPDCA^+^ cells (Figure S3C. Related to Figure 5). These data suggested that the STING signaling pathway mediated DC maturation and pDC differentiation.

### STING Activation Induced DC Maturation and Promoted the Interaction between LYN and STING in DC

**Figure 6:**
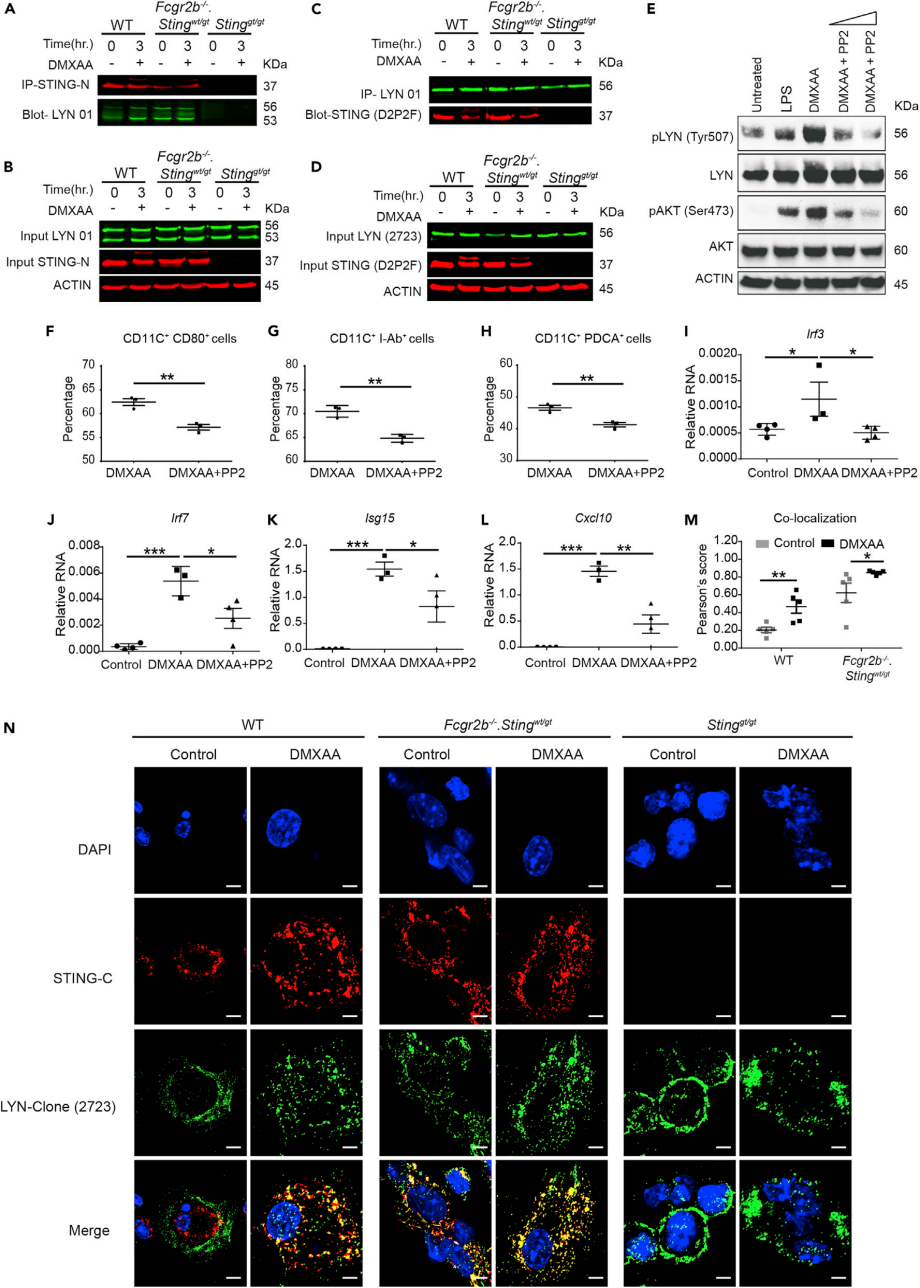
STING Activation Induced DC Maturation and Promoted the Interaction between LYN and STING in DC (A–D) Fluorescent western blot shows (A) the immunoprecipitation (IP) with STING-N (red) and blots with Lyn (green) and (B) cell lysate of activated BMDC with DMXAA at 0 and 3 h (C) A reverse IP using the Lyn antibody and blot with STING antibody and (D) cell lysate of activated BMDC with DMXAA at 0 and 3 h. Data show a representative of four experiments. (E–L) (E) Western blot analysis of Sting-activated BMDC with or without PP2 inhibitor showed the phosphorylation of Lyn (Try507) and Akt (Ser473). Data are representative of three mice per group. Sting-activated BMDCs were cultured with Lyn inhibitor (PP2) and analyzed by (F–H) flow cytometry shows the percentage of (F) CD80^+^CD11c^+^, (G) I-Ab^+^CD11c^+^, and (H) PDCA^+^CD11c^+^ cells (N = 3 per group), and (I–L) the relative RNA expression (normalized by actin) of (I) *Irf3*, (J) *Irf7*, (K) I*sg15*, and (L) *Cxcl10* are shown (N = 4 per group). (M and N) Confocal microscopy of DMXAA-activated BMDC from WT, *Sting*^*gt/gt*^, and *Fcgr2b*^*−/−*^*. Sting*^*wt/gt -*^ mice for 6 h. (M) The quantification of colocalization signals between STING and Lyn (N = 5 per group). Data are shown as mean ± SEM; ∗p < 0.05, ∗∗p < 0.01, and ∗∗∗p < 0.001. (N) Immunofluorescence staining of BMDC shows Lyn (green), STING (red), and DAPI (blue) (scale bar, 20 μm). Data show a representative of five experiments.

STING-interacting proteins were identified by immunoprecipitation (IP) using the STING antibody that targets the N-terminal region of the protein. STING-activating BMDC with DMXAA for 3 h was immunoprecipitated and analyzed by mass spectrometry (Table S2. Lists of STING interacting proteins. Related to Figure 6). Among the proteins detected, LYN, a member of Src family kinases, has been shown to function in the maturation of DC and pDC response (Chu and Lowell, 2005; Dallari et al., 2017). Immunoprecipitation confirmed that LYN interacted with STING after DMXAA stimulation in WT BMDC, whereas LYN constitutively interacted with STING in the *Fcgr2b*-deficient BMDC (Figures 6A and 6C). This interaction could result from the intrinsic activation of STING in the *Fcgr2b*^*−/−*^ mice. This activation did not change the protein abundance of LYN and STING in total cell lysate (Figures 6B and 6D). Also, the western blot from both IP and cell lysate showed another fainting band of STING after the activation; this protein was identified by mass spectrometry as the phosphorylation of STING (Ser357) (Figures S4A–S4B. Related to Figure 6). The activation of STING increased the phosphorylation of LYN (Tyr507) and AKT (Ser473), which were inhibited by PP2 (Figures 6E and S5D–S5E. Related to Figure 6).

Lyn kinase regulates DC maturation, and genetic deletion of *Lyn* ablates pDC (Chu and Lowell, 2005; Dallari et al., 2017). We hypothesized that STING promoted DC maturation and pDC differentiation through LYN signaling pathway, and the inhibition of LYN should affect the STING-induced BMDC differentiation. The *in vitro* data showed that pan SFK inhibitor PP2 decreased STING-mediated expression of IAb and CD80 on conventional DC (Figures 6F, 6G, and S4C. Related to Figure 6) and the differentiation of pDC (Figure 6H). Next, we tested whether PP2 inhibited STING-mediated signaling. The PP2 decreased the mRNA expression of *Irf3*, *Irf7*, *Isg15*, and *Cxcl10* in the STING-stimulated BMDC (Figures 6I–6L). These data suggested that PP2 treatment reduced the enhanced expression of ISG via STING activation.

To confirm the physical interaction between STING and LYN, we identified the colocalization of STING and LYN in the BMDC using two different clones of anti-STING and anti-LYN antibodies (Figures 6N and S5A. Related to Figure 6). The quantification of fluorescence signaling showed a significant increase in colocalization of STING and LYN upon STING stimulation (Figures 6M and S5C. Related to Figure 6). The *Fcgr2b*-deficient BMDC constitutively showed a certain degree of the colocalization between STING and LYN, whereas the activation of STING promoted more interaction (Figure 6N). Also, FYN, a member of the Src family kinases (SFKs), has been shown to have functional role in pDC maturation and PP2 inhibited both LYN and FYN signaling (Dallari et al., 2017). Therefore, we identified if FYN colocalized with STING and found that FYN did not colocalize with STING (Figure S5B. Related to Figure 6). These data suggested that activation of STING induced LYN interaction and mediated maturation and differentiation of conventional DCs and pDCs.

### Adoptive Transfer of *Sting*-Expressing BMDC Induces Lupus Development in the *Fcgr2b*^*−/−*^*.Sting*^*gt/gt*^ Mice

**Figure 7:**
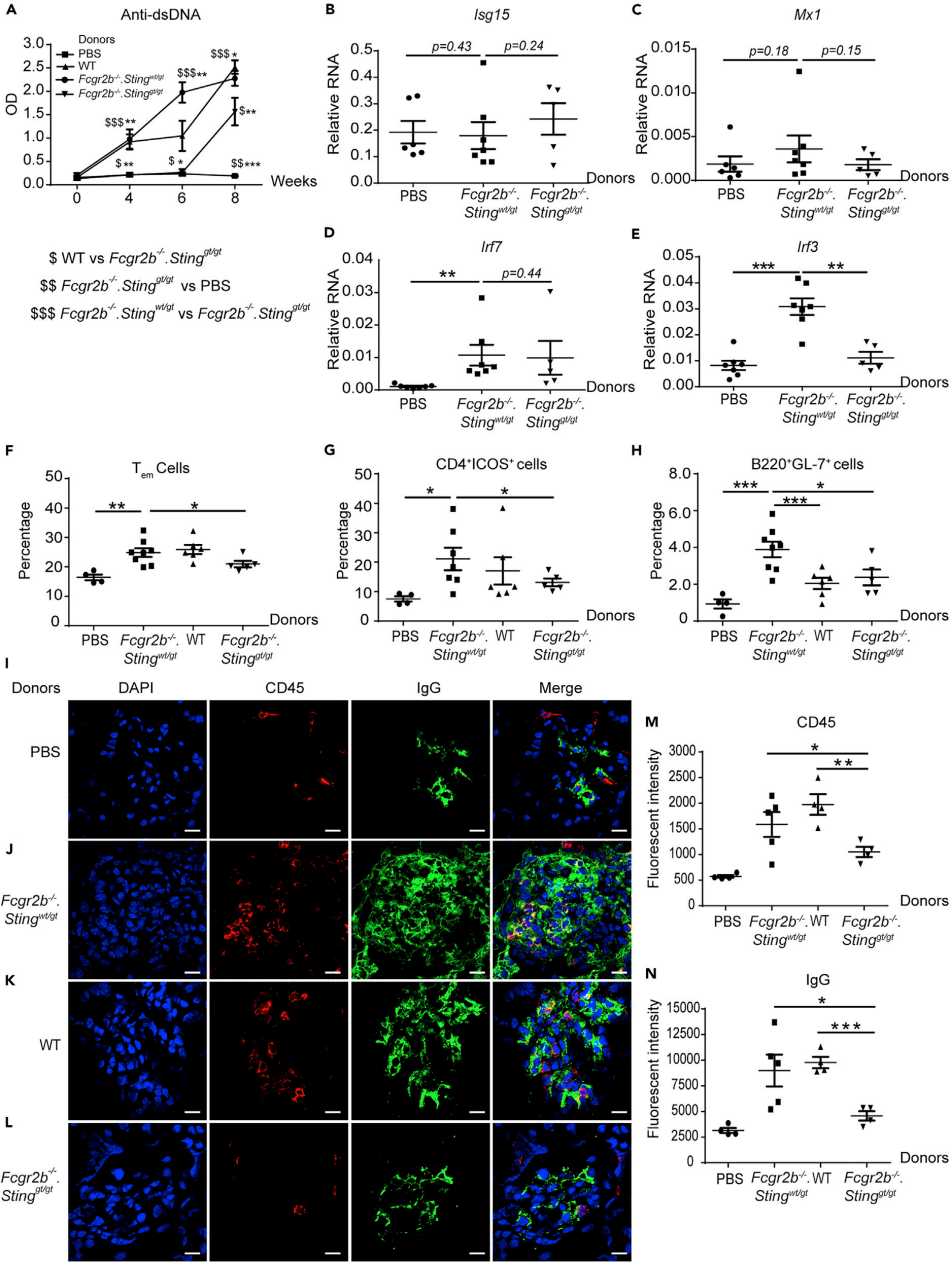
Adoptive Transfer of *Sting*-Expressing BMDC Induces Lupus Development in the *Fcgr2b*^*−/−*^.*Sting*^*gt/gt*^ Mice DMXAA-activated BMDC from *Fcgr2b*^*−/−*^.*Sting*^*wt/gt*^, WT, and *Fcgr2b*^*−/−*^.*Sting*^*gt/gt*^ were transferred into the recipient mice (*Fcgr2b*^*−/−*^.*Sting*^*gt/gt*^). (A) The level of anti-dsDNA from the sera (1:100) measured by ELISA (N = 5–10 per group). The dollar sign ($) shows the comparison between the groups. (B–E) The relative RNA expressions (normalized by actin) of (B) *Isg15*, (C) *Mx1*, (D) *Irf7*, and (E) *Irf3* in the kidney of the mice receiving BMDC are shown (N = 5–6 per group). (F–H) Flow cytometry analysis of recipient splenocytes after BMDC transferred every 2 weeks for four times shows the percentage of (F) T_em_ (CD4^+^CD44^hi^CD62L^lo^), (G) CD4^+^ICOS^+^ cells, (H) B220^+^GL7^+^ cells (N = 5–10 per group). (I–L) Immunofluorescence staining of the kidney from the *Fcgr2b*^*−/−*^.*Sting*^*gt/gt*^ recipient mice after the transfer with (I) PBS control, DMXAA-activated BMDC from (J) *Fcgr2b*^*−/−*^.*Sting*^*wt/gt*^, (K) WT, and (L) *Fcgr2b*^*−/−*^.*Sting*^*gt/gt*^. The confocal microscope shows DAPI (blue), CD45 (red), and IgG (green). The representative of four experiments (scale bar, 10 μm). (M and N) The quantification of fluorescence intensity of (M) CD45, and (N) IgG staining. Data are shown as mean ± SEM; ∗p < 0.05, ∗∗p < 0.01, and ∗∗∗p < 0.001.

The STING signaling pathway activated the immature BMDC to differentiate into the mature DC and pDC. The dendritic cells are significant producers of inflammatory cytokines and capable of promoting T cell proliferation and differentiation. We proposed that STING may induce the lupus disease by initially acting through the DC activation. We performed the adoptive transfer of the STING-activated BMDC derived from *Fcgr2b*^*−/−*^ mice into WT recipient mice to test this hypothesis. The recipient WT mice developed the autoimmune phenotypes, including the production of anti-dsDNA (Figure S6A. Related to Figure 7), expansion of Tem (Figure S6B. Related to Figure 7), CD4^+^ICOS^+^ (Figure S6C. Related to Figure 7), CD138^+^ (Figure S6D. Related to Figure 7), germinal center B cells (Figure S6E. Related to Figure 7), and minimal immune complex deposition in the kidneys (Figures S6F–S6G. Related to Figure 7). Although only minimal IgG deposition was detected, these data suggested that STING-activated BMDC can induce autoimmunity. The background of WT recipient mice may not promote the overt phenotypes of autoimmune disease.

Next, we performed the reconstitution experiment by adoptive transfer of STING-activated BMDC into the double-deficient mice. The level of anti-dsDNA significantly increased in the mice receiving *Sting*-sufficient BMDC compared with those receiving *Sting*-deficient BMDC and control (Figure 7A). The transfer of BMDC in the double-deficient mice did not increase the expression of *Isg15*, *Mx1*, and *Irf7* (Figures 7B–7D); however, *Irf3* increased in the kidney of the recipient mice (Figure 7E).

The analysis of spleens showed an increase in the percentage of T_em_ and CD4^+^ICOS^+^ in recipient mice that received STING-activated BMDC derived from WT or *Fcgr2b*^*−/−*^*.Sting*^*wt/gt*^ mice when compared with the PBS control and double-deficient BMDC injection group (Figures 7F and 7G). These data suggested that T cell phenotypes required STING expression in BMDC. Interestingly, only *Sting*-sufficient BMDC from *Fcgr2b*^−/-^, but not WT mice, induced the spontaneous germinal center B cell formation. Also, *Sting*-deficient BMDC from *Fcgr2b*^−/-^ did not increase germinal center B cell (Figure 7H). Next, we examined the immunofluorescence staining to identify the immune complexes at the kidney of the recipient. The recipient of *Sting*-sufficient BMDC showed an increase of IgG deposition and CD45^+^ cell infiltration, whereas *Sting*-deficient BMDC did not (Figures 7I–7N).

Nevertheless, the *Fcgr2b*^*−/−*^ BMDC induced more immune complexes and CD45^+^ cells in the kidneys (Figures 7J and 7K). The results suggested that the restoration of the STING signaling pathway in dendritic cells is essential for lupus development in the *Fcgr2b*^*−/−*^*.Sting*^*gt/gt*^ mice. The *Sting*-sufficient BMDC induced the lupus phenotypes in the double-deficient mice via the activation of T and B cells, which led to autoantibody production rather than promote type I IFN signaling.

## Discussion

A gain-of-function mutation in STING has been identified as a gene responsible for a subpopulation of patients with SLE, and STING-dependent interferon-inducible genes correlated with disease activity (Kato et al., 2018; Konig et al., 2017). However, there are no functional data of STING in a lupus mouse model that are relevant to human SLE. The 129/B6.*Fcgr2b*^*−/−*^ mice carrying Nba2 region expressed constitutively *Ifi202*. The CD19^+^ cells from B6.Nba2 show the increase of *Ifi202* and the decrease of *Sting* expression (Panchanathan et al., 2013). However, the overexpression of *Ifi202* can activate the Sting-dependent IFN-I response and the 129/B6.*Fcgr2b*^*−/−*^ mice increase the expression of IFN-β (Brunette et al., 2012; Panchanathan et al., 2011). Here, we detected the high expression of *Sting*, *Ifn-β*, and interferon-inducible genes from the spleen of these mice. The activation of cGAS leads to cGAMP production and subsequently activates STING signaling (Sun et al., 2013; Wu et al., 2013). The increase of cGAMP in the 129/B6.*Fcgr2b*^*−/−*^ mice raised the question of the natural origin of DNA that stimulates the cGAS-STING pathway. The *Fcgr2b*^*−/−*^ mice showed the cGAMP overproduction, whereas the disruption of STING decreased the level of cGAMP in the *Fcgr2b*^*−/−*^ mice. On the contrary to a previous study, the increase of cGAMP in *Trex1* and *DnaseII*-deficient mice was upregulated in the absence of STING (Gao et al., 2015). TREX1 degrades double-stranded DNA, whereas oxidized DNA is resistant to TREX1-mediated degradation (Gehrke et al., 2013; Grieves et al., 2015). Also, DNASE II cleaves native dsDNA but works less effectively for denaturized DNA (Laukova et al., 2020). Moreover, the mitochondrial DNA (mtDNA) releasing into the cytosol can trigger the IFN-I response and accelerates the severity of a mouse model of lupus disease (Kim et al., 2019). The mtDNA released into the cytosol activates the cGAS-STING pathway in cisplatin-induced tubular inflammation (Maekawa et al., 2019). Since the absence of STING reduced the level of cGAMP in the *Fcgr2b*^*−/−*^ mice, but not in the *Trex1*^*−/−*^ and *DnaseII*^*−/−*^ mice, the intrinsic DNA origin that constitutively activates the STING-cGAS pathway should be different among these autoimmune mouse models. The *Fcgr2b*^*−/−*^*.Sting*^*gt/gt*^ mice did not develop overt kidney inflammation; thus, it is likely to release less amount of oxidized mtDNA. The cytosolic DNA that constitutively activates STING-cGAS pathway in the *Fcgr2b*^*−/−*^ mice may originate from stress-induced mitochondrial DNA leakage from inflammation. These findings suggested that specific types of DNA from different compartments could be sensed and activated through the STING-cGAS pathway.

Although STING functions as a negative regulator in the Mrl/*lpr* lupus mice (Sharma et al., 2015), our data show that STING is required for the lupus development in the 129/B6.*Fcgr2b*^*−/−*^ mice. STING may also play a crucial role in other lupus mouse models, which contained the Nba2 region. The survival of the 129/B6.*Fcgr2b*^*−/−*^ mice depend on autoantibody production and glomerulonephritis (Bolland et al., 2002; Pisitkun et al., 2012). STING is required for the antibody production induced by cyclic-di-GMP *in vitro* (Walker et al., 2018). These data suggested that STING facilitated the autoantibody production, inflammatory cell infiltration, and glomerulonephritis in the 129/B6.*Fcgr2b*^*−/−*^ mice. The expression of interferon-inducible genes associated with SLE disease activity (Feng et al., 2006). We detected the very high expression of IFN-inducible genes in the kidneys of 129/B6.*Fcgr2b*^*−/−*^ mice showed severe pathology. The absence of STING signaling in the *Fcgr2b*^*−/−*^ mice partly decreased the expression of interferon-inducible genes in the kidney. The kidneys in the experiment were not perfused; thus, we cannot conclude that the IFN signature expressed in the kidney derived from the blood or kidney. These data suggested that other nucleic acid sensors may promote the type I interferon production or signaling in the *Fcgr2b*^*−/−*^ mice as well, and STING-dependent lupus phenotypes do not mediate only through type-I interferon pathway.

STING expresses and functions differentially depending on the cell types. STING signals synergistically with B cell receptor signaling to promote antibody response (Walker et al., 2018). Our results showed that spontaneous germinal center B cells and MHC-II expression in the *Fcgr2b*^*−/−*^ mice were *Sting* dependent. However, plasma cell expansion was *Sting* independent. These data suggested STING may contribute to the autoantibody production through memory B cells. STING also activates T cells, which induced type I IFN production and mediated cell death (Larkin et al., 2017). Nevertheless, we found that the increase of T effector memory (T_em_) in the *Fcgr2b*^*−/−*^ mice was *Sting* dependent. The expansion of T_em_ may directly mediate through the interaction with antigen-presenting cells, not via Sting signaling in T cells.

STING agonist (DMXAA)-treated mice show the increased expression of CD80, CD86, and MHC-II on DC, suggesting the mature phenotypes of DC as the antigen-presenting cells (APCs) (Curran et al., 2016). We observed the reduction of DC expansion in the *Fcgr2b*^*−/−*^ mice, which depended on STING signaling. We confirmed that STING was required for DC maturation and cytokine production. These DCs became professional APCs and could promote T cell differentiation. The IFN-γ-producing CD4^+^ cells in the spleen of the *Fcgr2b*^*−/−*^ mice were reduced in the absence of STING. The *Sting*-expressing DCs derived from WT and *Fcgr2b*^*−/−*^ mice stimulated naive T cells to proliferate; however, the ability of T cells to differentiate and produce IFN-γ did not depend on intrinsic *Sting* expression on T cells. Interestingly, only DCs from the *Fcgr2b*^*−/−*^ mice can increase the IFN-γ production in CD4^+^ T cells. These data suggested the DC from the *Fcgr2b*^*−/−*^ mice have the intrinsic property that promotes the generation of IFN-γ-producing CD4^+^ T cells.

The cGAS-STING signaling can activate human pDCs to produce IFN-I, and knockdown of *Sting* using siRNA in CAL-1 cells can cause the reduction of IFN response (Bode et al., 2016). The proteomic data showed the upregulation of interferon-regulated protein after STING activation with DMXAA, which implied that the culture environment should enrich with type-I IFN. STING activation led to phosphorylation of Ser357 of mouse Sting (homolog Ser358 in human Sting), and this site is phosphorylated by TBK1 which subsequently promoting type I IFN production (Tanaka and Chen, 2012; Zhong et al., 2008). The identification of pDC after STING activation uncovered the role of STING in the differentiation of pDC. These data revealed that STING was essential for the generation of pDCs. Besides, our study identified several STING-interacting proteins by mass spectrometry. Lyn kinase has been shown to have role in the differentiation of pDC (Dallari et al., 2017). The recruitment of LYN to STING after DMXAA stimulation suggested STING-mediated signaling through Lyn kinase. Also, the proteomics data of STING-activated BMDC showed a significant increase of phosphoinositide 3-kinase adapter protein 1 (Pik3ap1) and receptor of activated protein C kinase 1 (Rack1) (Table S1. Lists of up- and down-regulated proteins. Related to Figure 5). Pik3ap1 is an adaptor that signals to the phosphoinositide 3-kinase (PI3K) (Aiba et al., 2008). LYN and RACK1 are co-immunoprecipitated in membrane complexes (Sutton et al., 2013). Also, RACK1 silencing affected the phosphorylation of AKT (Liu et al., 2017). Our data suggest that the downstream of STING-LYN signaling mediated through the PI3K-AKT pathway. The inhibition of Lyn kinase with PP2 inhibitor during STING activation diminished DC maturation and pDC differentiation. The PP2 also is a broad inhibitor of other Src kinase family. However, we could identify the interaction of LYN and STING by mass spectrometry, immunoprecipitation, and colocalization during STING activation. All of these data suggested STING-mediated differentiation of BMDC probably through the LYN signaling pathway.

The duration of the transfer experiment was 8 weeks. Thus, the phenotypes that have changed would not be apparent lupus, but still, the hallmarks of lupus disease settled down in the recipient mice. However, the increase of anti-dsDNA in the serum, immune complex deposition, and inflammatory cell infiltration in the kidney were detected in the recipient mice when transferring the STING-sufficient BMDC into the double-deficient recipient mice.

The depletion of pDC ameliorates the autoimmune phenotypes in BXSB lupus-prone mice and B6.Nba2 mice (Davison and Jørgensen, 2015; Rowland et al., 2014). Our data strongly suggested STING involving in DC function both DC maturation and pDC differentiation. Adoptive transfer of *Sting*-sufficient BMDCs can induce autoantibody production and immune complex deposition regardless of the *Fcgr2b* status. However, the absence of *Fcg2b* in the BMDC can accelerate the severity of autoimmune phenotypes and notably increase inflammatory cell infiltration in the kidney of the double-deficient recipient mice.

The transfer of *Sting*-sufficient *Fcgr2b*^*−/−*^ BMDC led to an increase of *Irf3* expression but not *Isg15, Mx1, and Irf7.* Irf3 is a transcription factor downstream of STING signaling pathway (Burdette et al., 2011). These data suggested that *Irf3* expression in the kidney was STING-dependent BMDC, whereas other ISGs may depend on the intrinsic STING expression of the resident cells in the kidney. Besides, the transfer of *Sting*-sufficient *Fcgr2b*^*−/−*^BMDC could increase the effector T cell and germinal center B cells in the spleens of the recipient mice. These data suggested that STING expression on BMDC is essential for the initiation of autoimmunity in the double-deficient mice via the activation of T and B cells, which led to autoantibody production rather than the promotion of type I IFN signaling.

In summary, this study established the vital function of STING in the autoimmune *Fcgr2b*^*−/−*^ lupus mouse model, thus providing a reliable tool for future mechanistic and preclinical studies of STING in SLE. These findings provide proof of the concept that inhibition of STING signaling may be a candidate targeted treatment for a subset of patients with SLE.

### Limitations of the Study

This study identified the functional role of LYN in the differentiation of BMDC based on the PP2 inhibitor, which is a broad inhibitor for the Src kinase family. The *Lyn*-deficient BMDC will be solid proof to conclude that STING mediated DC differentiation via LYN kinase. However, the identification of LYN that immunoprecipitated with STING in BMDC by mass spectrometry but not other members of the Src family suggested the interaction of LYN and STING in the BMDC. Furthermore, we showed the vital role of STING in the *Fcgr2b*-deficient mice, one of the lupus mice models. The study of STING function in the human SLE in the future research would suggest the promising target to inhibit STING signaling in the treatment of SLE.

### Resource Availability

#### Lead Contact

Further information and requests for resources and reagents should be directed to the Lead Contact, Dr. Prapaporn Pisitkun (Prapaporn.pis@mahidol.ac.th).

#### Materials Availability

Materials are available from the corresponding author on a reasonable request.

#### Data and Code Availability

The microarray data are available at Gene Expression Omnibus: GSE142594 and https://www.ncbi.nlm.nih.gov/geo/query/acc.cgi?acc=GSE142594.

The mass spectrometry proteomics data have been deposited to the ProteomeXchange Consortium via the PRoteomics IDEntifications (PRIDE) partner repository with the dataset identifier PXD019239.

## Methods

All methods can be found in the accompanying Transparent Methods supplemental file.

## Supplemental Information

Document S1. Transparent Methods, Figures S1–S6, and Table S2

Table S1. Lists of Up and Down of Regulated Proteins, Related to Figure 5
