# Supplementary material for: STING Mediates Lupus via the Activation of Conventional Dendritic Cell Maturation and Plasmacytoid Dendritic Cell Differentiation
Source: iScience. 2020 Sep 4;23(9):101530. doi: 10.1016/j.isci.2020.101530 (PMC7502826; doi:10.1016/j.isci.2020.101530)
Supplement: Document S1. Transparent Methods, Figures S1–S6, and Table S2 [file mmc1.pdf]

iScience, Volume *23*

## Supplemental Information

# STING Mediates Lupus via the Activation of Conventional Dendritic Cell Maturation and Plasmacytoid Dendritic Cell Differentiation

**Arthid Thim-uam, Thaneas Prabakaran, Mookmanee Tansakul, Jiradej Makjaroen, Piriya Wongkongkathep, Naphat Chantaravisoot, Thammakorn Saethang, Asada Leelahavanichkul, Thitima Benjachat, Søren Paludan, Trairak Pisitkun, and Prapaporn Pisitkun**

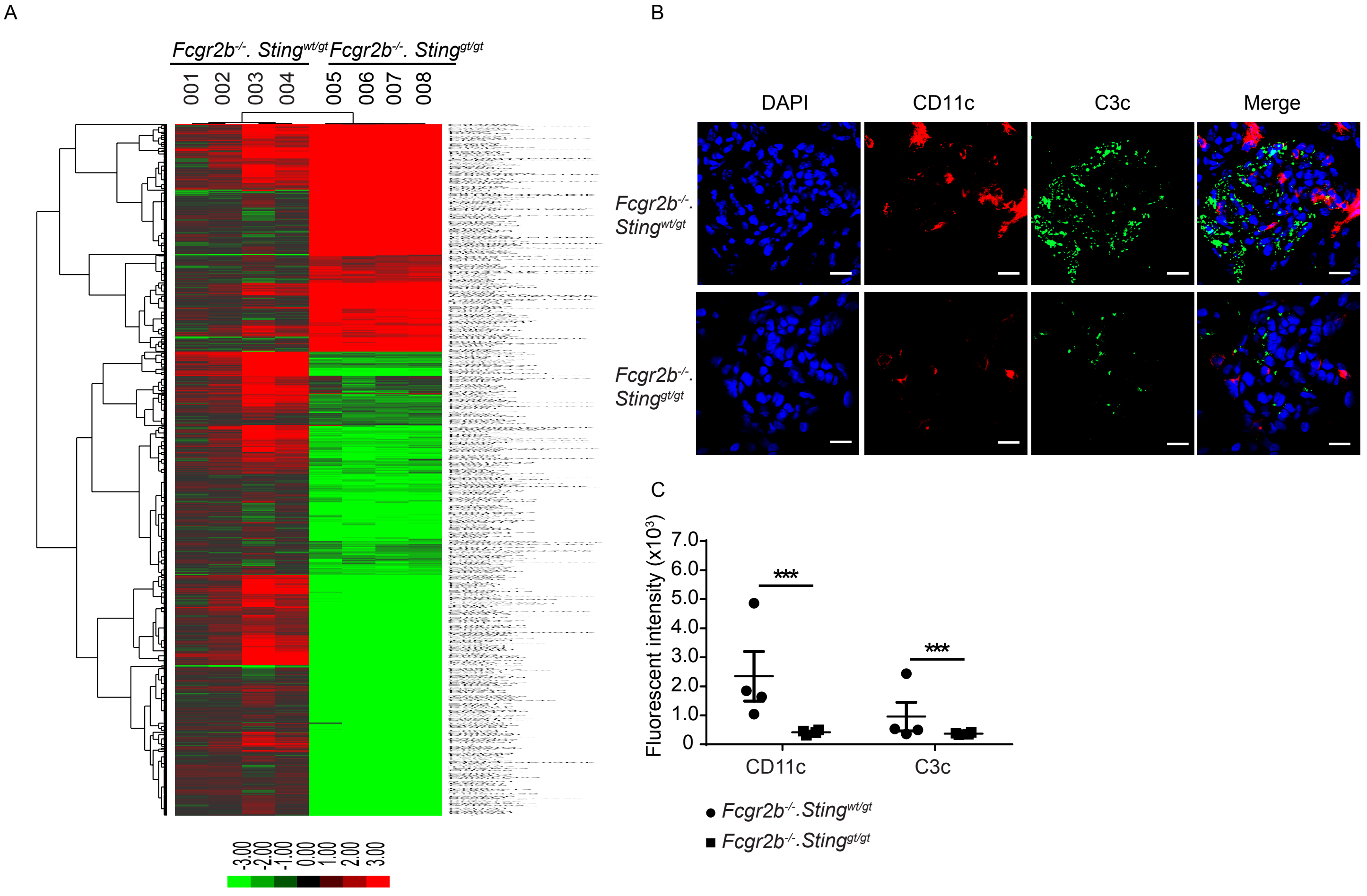


**Figure S1. Phenotypes of kidney in the Fcgr2b$^{-/-}$. Sting$^{wt/gt}$ and Fcgr2b$^{-/-}$. Sting$^{gt/gt}$ mice at the age of 6-8 months, Related to Figure 2.** (A) Genes expression profiles of kidneys in the *Fcgr2b$^{-/-}$. Sting$^{wt/gt}$* and *Fcgr2b$^{-/-}$. Sting$^{gt/gt}$* mice. A heat map of microarray data shows the genes that significantly changed up to 2 fold compared between *Fcgr2b$^{-/-}$. Sting$^{wt/gt}$* and *Fcgr2b$^{-/-}$. Sting$^{gt/gt}$* mice (N=4 mice per group; p<0.05). Data show in log2 (sample/wild-type). (B) Immunofluorescence staining of the kidneys from *Fcgr2b$^{-/-}$. Sting$^{wt/gt}$* and *Fcgr2b$^{-/-}$. Sting$^{gt/gt}$* mice shows C3c (green), CD11c (red), and DAPI (blue). Data are representative of 4 mice per group (scale bar=10 μm). (C) The quantification of fluorescence intensity of CD11c and C3c was analyzed by ZEISS ZEN Microscope Software (Carl Zeiss, Germany) (N=4 mice per group). Data show as mean ± SEM; *p < 0.05, **p<0.01 and ***p<0.001.1).

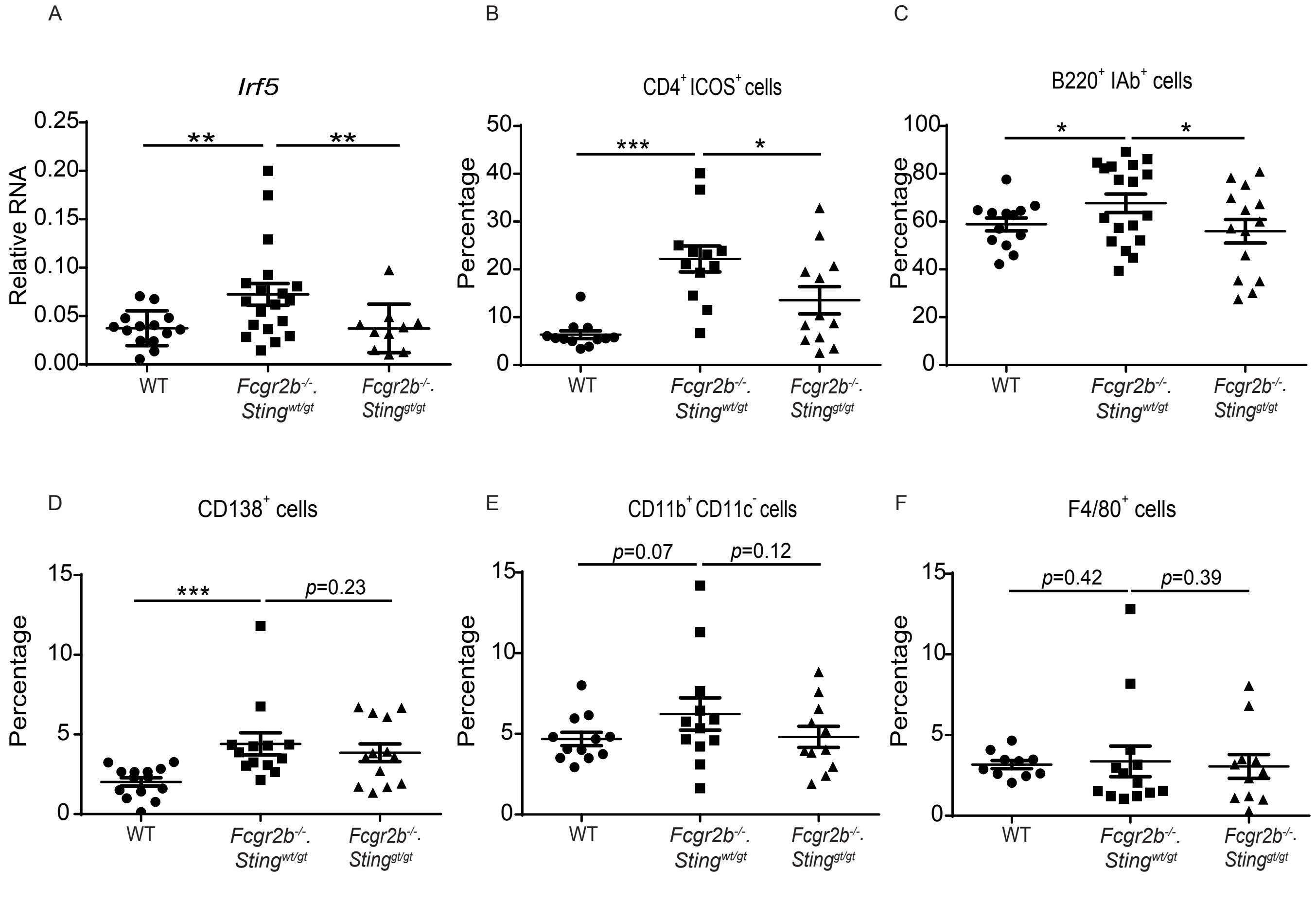


**Figure S2. STING signaling is essential for immuno-phenotypes of the Fcgr2b$^{-/-}$. Sting$^{wt/gt}$ lupus mice,Related to Figure 3.**
(A) Gene expression from the kidneys of WT, *Fcgr2b$^{-/-}$. Sting$^{wt/gt}$* and *Fcgr2b$^{-/-}$. Sting$^{gt/gt}$* mice at the age of 6 months were tested by real-time PCR. The relative RNA (normalized by actin) of (A) *Irf5* is shown (N=10-17 per group). (B-F) Flow cytometry analysis of splenocytes isolated from WT, *Fcgr2b$^{-/-}$. Sting$^{wt/gt}$* and *Fcgr2b$^{-/-}$. Sting$^{gt/gt}$* mice at the age of 6-7 months (N= 13-14 per group). Data shown in the percentage of (B) CD4$^+$ ICOS$^+$ cells, (C) B220$^+$ I-Ab$^+$ cells, (D) CD138$^+$ cells, (E) CD11b$^+$ CD11c$^-$ cells and (F) F4/80$^+$ cells. Data show as mean ± SEM (*p < 0.05, **p<0.01 and ***p<0.001).

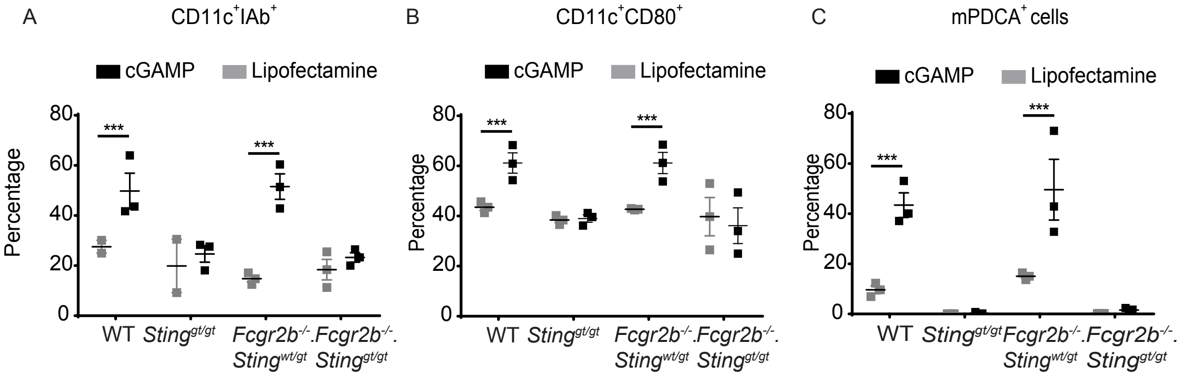


**Figure S3. STING activation promotes the maturation of dendritic cells and the differentiation of plasmacytoid dendritic cells, Related to Figure 5.** Bone marrows were isolated from WT, $Sting^{gt/gt}$, $Fcgr2b^{-/-}$ . $Sting^{wt/gt}$ and $Fcgr2b^{-/-}$. $Sting^{gt/gt}$ mice atthe age of 6 months. (A-C) Bone marrow-derived dendritic cells were differentiated with IL-4 and GM-CSF differentiated for five days and subsequently stimulated with cGAMP for 24 hours. Flow cytometry analysis shows the percentage of (A) $CD11c^{+}$ $IAb^{+}$ cells and (B) $CD11c^{+}CD80^{+}$ cells, and (C) $PDCA^{+}$ cells after cGAMP activation (N=3). Data show as mean ± SEM; *p < 0.05, **p<0.01 and ***p<0.001.

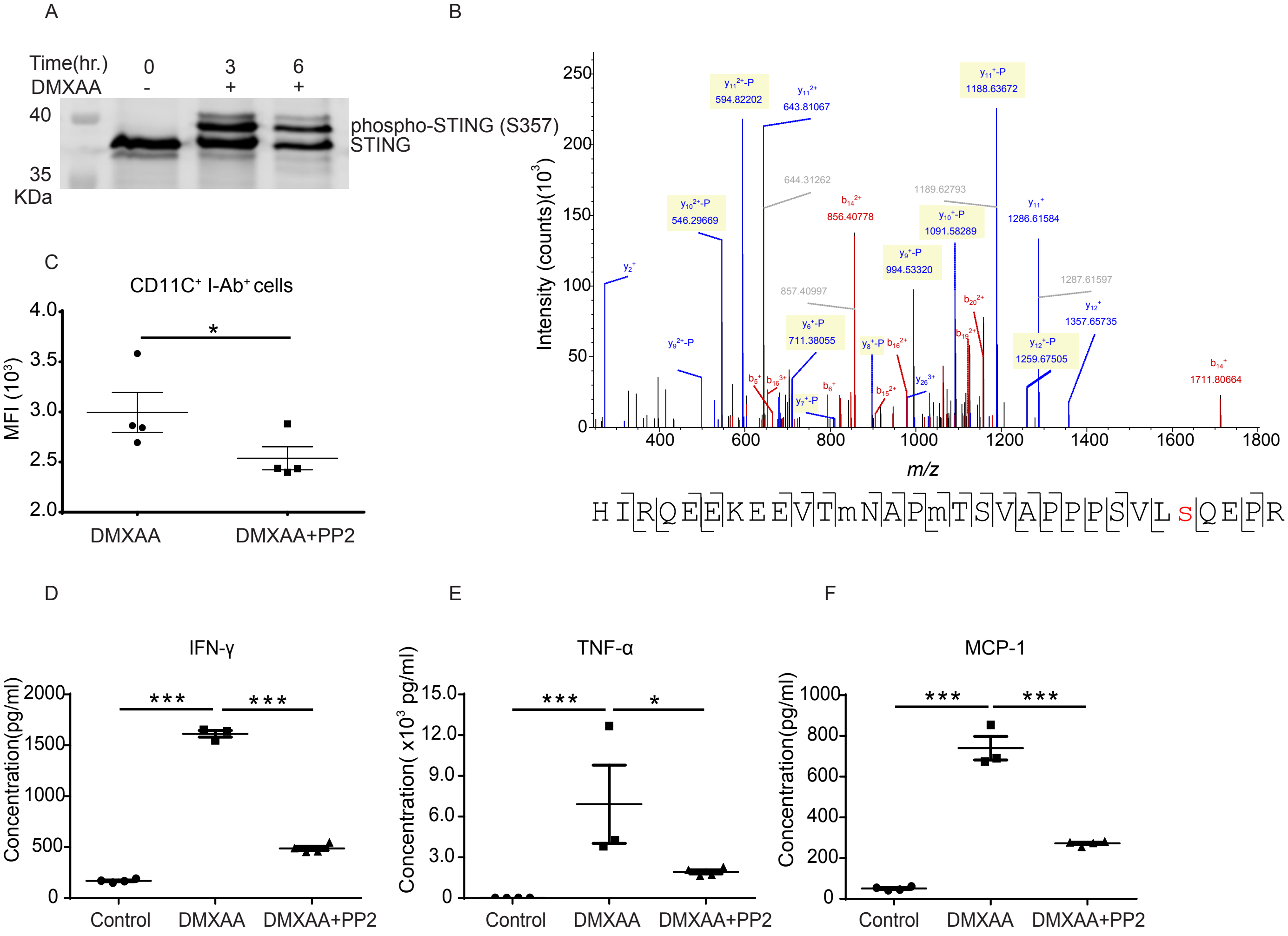


**Figure S4. Phenotypes of STING activated dendritic cells, Related to Figure 6.** (A) Representative of western blot analysis from immunoprecipitation with STING of *Fcgr2b*$^{-/-}$. *Sting*$^{wt/gt}$ mice (N= 4). The BMDC was activated with DMXAA at 0, 3, and 6 hours. The band is shown in STING protein and phosphorylation of STING at Ser357. (B) Mass spectra of phosphorylation of STING at Ser357 of activated BMDC from *Fcgr2b*$^{-/-}$. *Sting*$^{wt/gt}$ mice after stimulated with DMXAA for 3 hours and followed by immunoprecipitation with Sting. (C) Sting-activated BMDC were co-cultured with PP2 (Lyn inhibitor) and analyzed by flow cytometry. The mean fluorescence intensity (MFI) of IAb expressing DC from *Fcgr2b*$^{-/-}$. *Sting*$^{wt/gt}$ mice is shown (N = 4 mice per group). Data show as mean ± SEM; $*p < 0.05$. (D-F) Supernatants were collected and analyzed after DMXAA stimulation with or without PP2 inhibitor for 3 hours. Cytokines in the supernatant were detected by cytometric bead array shows the levels of (D) IFN-γ, (E) TNF-α and (F) MCP-1 (N=3-4). Data show as mean ± SEM; $*p < 0.05$, $**p<0.01$ and $***p<0.001$.

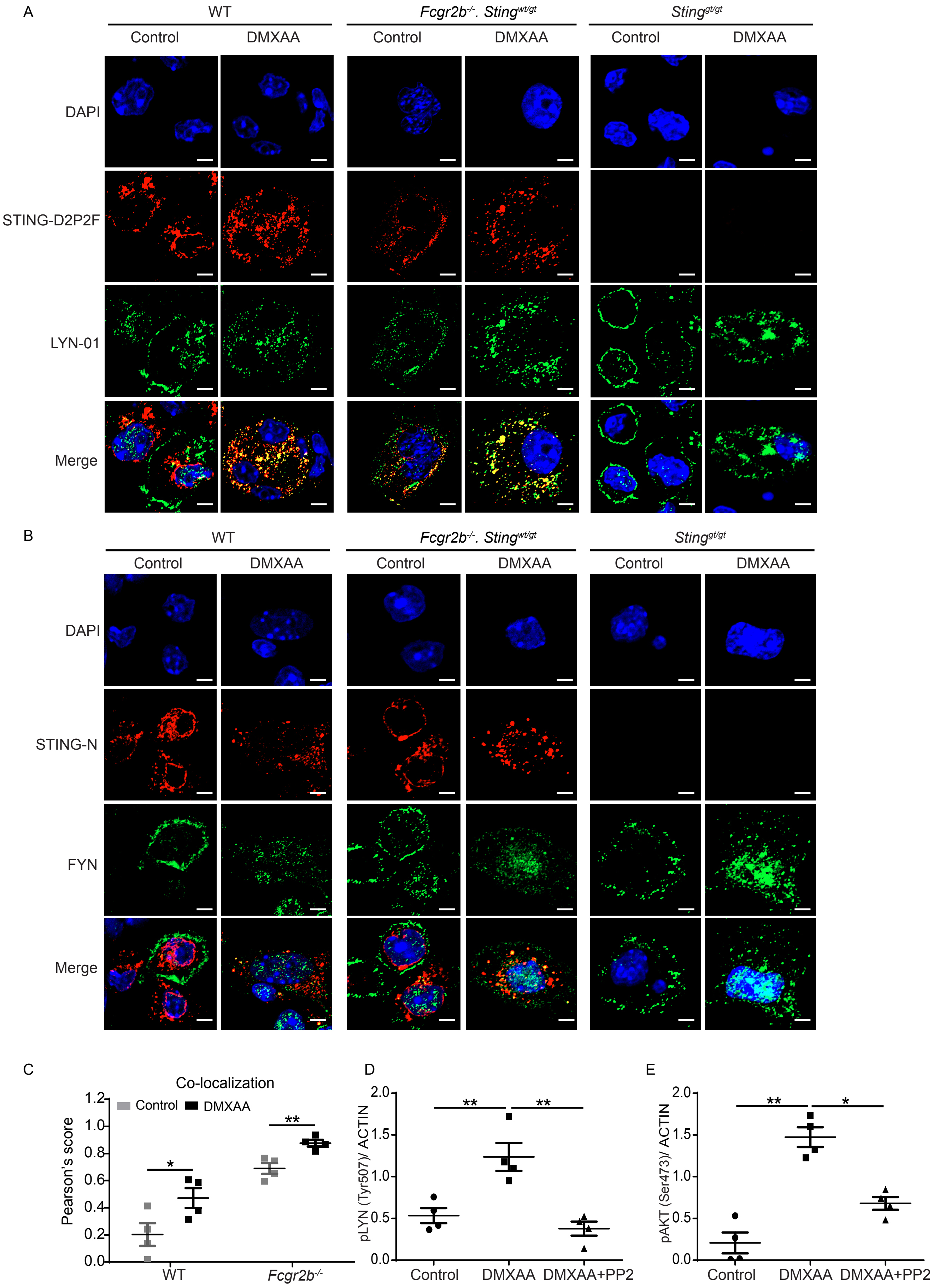


**Figure S5. STING activation induces the co-localization with LYN in the differentiation of BMDC, Related to Figure 6.** (A-B) The confocal microscope of DMXAA activated BMDC from WT, *Fcgr2b*$^{-/-}$. *Sting*$^{wt/gt}$ and *Sting*$^{gt/gt}$ mice for 6 hours. (A) Staining BMDC with Lyn (01) in green, STING-C (D2P2F) in red, and DAPI in blue is shown. (B) Staining BMDC with Fyn in green, Sting-N in red, and DAPI in blue (scale bar=20 um). The representative of 5 experiments is shown. (C) Co-localization score of STING and LYN were analyzed by ZEISS ZEN Microscope Software (Carl Zeiss, Germany) (N=4 mice per group). (D) Quantification of western blot of (D) pLYN (Tyr507) and (E) pAKT (Ser473) were analyzed by ImageJ software. Data show as mean± SEM; *p < 0.05, **p<0.01, and ***p<0.001.

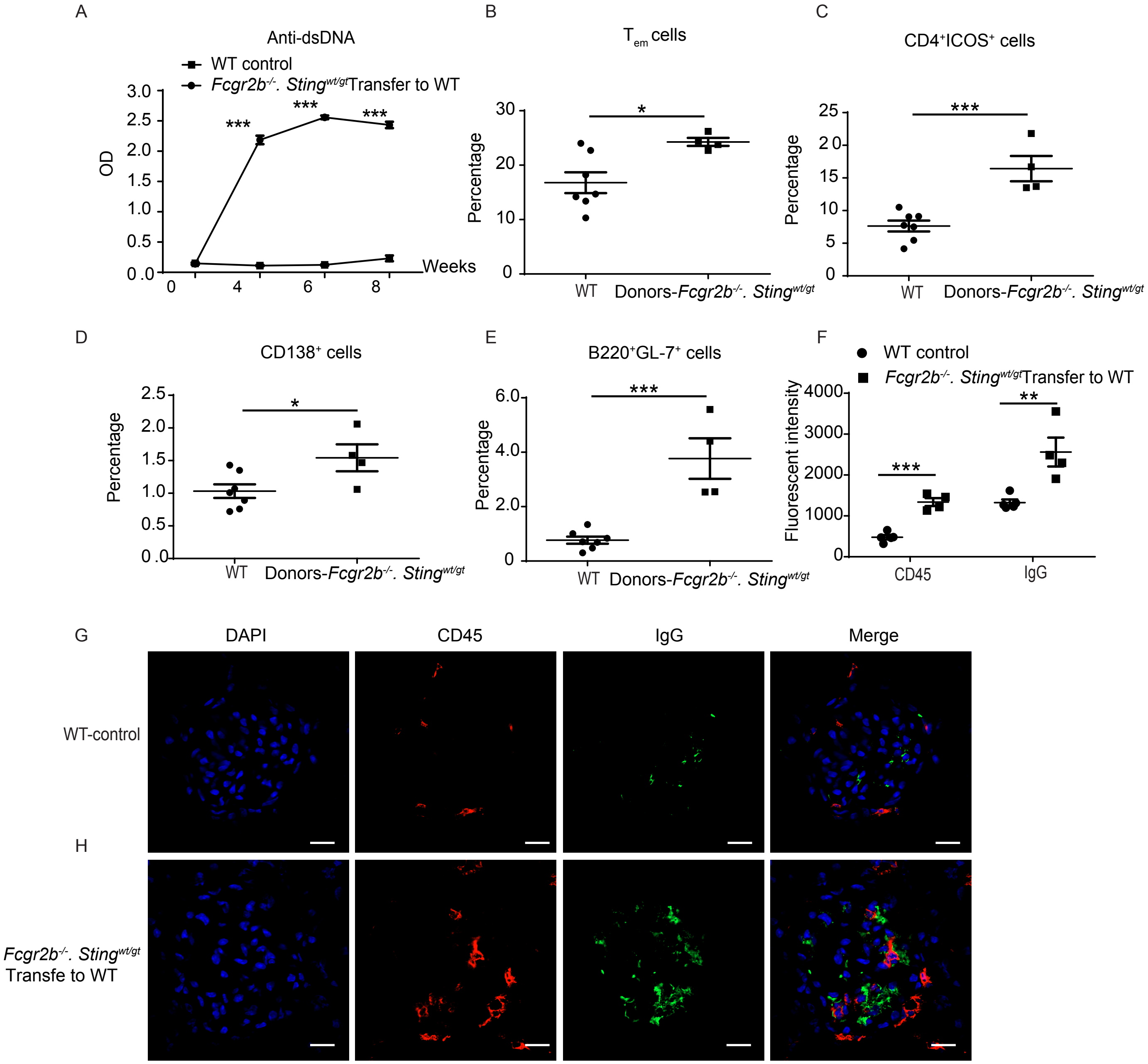


**Figure S6. Adoptive transfer of Sting expressing BMDC induces autoimmunity in WT mice, Related to Figure 7.** Transfer of DMXAA activated BMDC from *Fcgr2b*$^{-/-}$. *Sting*$^{wt/gt}$ were transferred into the recipient WT mice. (A) The level of anti-dsDNA from the sera (1:100) measured by ELISA (N=4-5 per group) were tested every 2 weeks after transfer. (B-E) Flow cytometry analysis of recipient splenocytes after BMDC transferred every 2 weeks for 4 times show the percentage of (B) effector T cells (CD4$^{+}$CD44$^{hi}$CD62L$^{lo}$), (C) CD4$^{+}$ICOS$^{+}$ cells, (D) CD138$^{+}$ cells, and (E) B220$^{+}$GL7$^{+}$ cells (N=4-7 per group). (F) Quantification of fluorescence intensity of CD45 and IgG were analyzed by ZEISS ZEN Microscope Software (Carl Zeiss, Germany) (N=4-5 mice per group). Data show as mean ± SEM; *p < 0.05, **p<0.01 and ***p<0.001. (G-H) Immunofluorescence staining on the kidneys of IgG in green, CD45 in red, and DAPI in blue from (G) non-transferred WT mice and (H) Fcgr2b$^{-/-}$. Sting$^{wt/gt}$ recipient WT mice are shown. Data are representative of 4-5 mice per group (scale bar=10 μm).

**Table S2. Lists of STING interacting proteins, Related to Figure 6**

| Accession | Gene name | Description |
|---|---|---|
| P60710 | Actb | Actin, cytoplasmic 1 [OS=Mus musculus] |
| Q922U2 | Krt5 | Keratin, type II cytoskeletal 5 [OS=Mus musculus] |
| P50446 | Krt6a | Keratin, type II cytoskeletal 6A [OS=Mus musculus] |
| Q6IME9 | Krt72 | Keratin, type II cytoskeletal 72 [OS=Mus musculus] |
| Q9Z2K1 | Krt16 | Keratin, type I cytoskeletal 16 [OS=Mus musculus] |
| P05213 | Tuba1b | Tubulin alpha-1B chain [OS=Mus musculus] |
| P21956 | Mfge8 | Lactadherin [OS=Mus musculus] |
| Q8BFR5 | Tufm | Elongation factor Tu, mitochondrial [OS=Mus musculus] |
| Q3UV17 | Krt76 | Keratin, type II cytoskeletal 2 oral [OS=Mus musculus] |
| Q3TBT3 | Tmem173 | Isoform 2 of Stimulator of interferon genes protein [OS=Mus musculus] |
| P20152 | Vim | Vimentin [OS=Mus musculus] |
| P13020 | Gsn | Gelsolin [OS=Mus musculus] |
| Q3TRJ4 | Krt26 | Keratin, type I cytoskeletal 26 [OS=Mus musculus] |
| O35744 | Chil3 | Chitinase-like protein 3 [OS=Mus musculus] |
| Q9QWL7 | Krt17 | Keratin, type I cytoskeletal 17 [OS=Mus musculus] |
| P01027 | C3 | Complement C3 [OS=Mus musculus] |
| P62631 | Eef1a2 | Elongation factor 1-alpha 2 [OS=Mus musculus] |
| E9Q557 | Dsp | Desmoplakin [OS=Mus musculus] |
| P62737 | Acta2 | Actin, aortic smooth muscle [OS=Mus musculus] |
| Q8C669 | Peli1 | E3 ubiquitin-protein ligase pellino homolog 1 [OS=Mus musculus] |
| P01029 | C4b | Complement C4-B [OS=Mus musculus] |
| Q3UHH1 | Zswim8 | Zinc finger SWIM domain-containing protein 8 [OS=Mus musculus] |
| P47856 | Gfpt1 | Glutamine--fructose-6-phosphate aminotransferase [isomerizing] 1 [OS=Mus musculus] |
| P70248 | Myo1f | Unconventional myosin-If [OS=Mus musculus] |
| Q5SUA5 | Myo1g | Unconventional myosin-Ig [OS=Mus musculus] |
| Q9WTI7 | Myo1c | Unconventional myosin-Ic [OS=Mus musculus] |
| P57780 | Actn4 | Alpha-actinin-4 [OS=Mus musculus] |
| Q9CZU3 | Mtrex | Superkiller viralicidic activity 2-like 2 [OS=Mus musculus] |
| Q9CYA6 | Zcchc8 | Zinc finger CCHC domain-containing protein 8 [OS=Mus musculus] |
| Q9QXS1 | Plec | Plectin [OS=Mus musculus] |
| Q8VDD5 | Myh9 | Myosin-9 [OS=Mus musculus] |
| P62830 | Rpl23 | 60S ribosomal protein L23 [OS=Mus musculus] |
| P01942 | Hba | Hemoglobin subunit alpha [OS=Mus musculus] |
| Q9WV32 | Arpc1b | Actin-related protein 2/3 complex subunit 1B [OS=Mus musculus] |
| P62874 | Gnb1 | Guanine nucleotide-binding protein G(I)/G(S)/G(T) subunit beta-1 [OS=Mus musculus] |
| P62908 | Rps3 | 40S ribosomal protein S3 [OS=Mus musculus] |
| Q9CPR4 | Rpl17 | 60S ribosomal protein L17 [OS=Mus musculus] |
| Q9CYL5 | Glipr2 | Golgi-associated plant pathogenesis-related protein 1 [OS=Mus musculus] |
| P47757 | Capzb | Isoform 3 of F-actin-capping protein subunit beta [OS=Mus musculus] |
| P25444 | Rps2 | 40S ribosomal protein S2 [OS=Mus musculus] |
| P60867 | Rps20 | 40S ribosomal protein S20 [OS=Mus musculus] |
| P07356 | Anxa2 | Annexin A2 [OS=Mus musculus] |

| Accession | Gene name | Description |
|---|---|---|
| Q6ZWN5 | Rps9 | 40S ribosomal protein S9 [OS=Mus musculus] |
| P35700 | Prdx1 | Peroxiredoxin-1 [OS=Mus musculus] |
| P15331 | Prph | Isoform 3u of Peripherin [OS=Mus musculus] |
| P62702 | Rps4x | 40S ribosomal protein S4, X isoform [OS=Mus musculus] |
| Q8CGP2 | Hist1h2bp | Isoform 2 of Histone H2B type 1-P [OS=Mus musculus] |
| Q60765 | Atf3 | Cyclic AMP-dependent transcription factor ATF-3 [OS=Mus musculus] |
| Q3V132 | Slc25a31 | ADP/ATP translocase 4 [OS=Mus musculus] |
| P58137 | Acot8 | Acyl-coenzyme A thioesterase 8 [OS=Mus musculus] |
| P62267 | Rps23 | 40S ribosomal protein S23 [OS=Mus musculus] |
| P59999 | Arpc4 | Actin-related protein 2/3 complex subunit 4 [OS=Mus musculus] |
| P14131 | Rps16 | 40S ribosomal protein S16 [OS=Mus musculus] |
| P08752 | Gnai2 | Guanine nucleotide-binding protein G(i) subunit alpha-2 [OS=Mus musculus] |
| Q64444 | Ca4 | Carbonic anhydrase 4 [OS=Mus musculus] |
| Q9DC51 | Gnai3 | Guanine nucleotide-binding protein G(k) subunit alpha [OS=Mus musculus] |
| Q9D8B3 | Chmp4b | Charged multivesicular body protein 4b [OS=Mus musculus] |
| Q8VED5 | Krt79 | Keratin, type II cytoskeletal 79 [OS=Mus musculus] |
| P11928 | Oas1a | 2'-5'-oligoadenylate synthase 1A [OS=Mus musculus] |
| Q9R0N7 | Syt7 | Isoform 4 of Synaptotagmin-7 [OS=Mus musculus] |
| P99024 | Tubb5 | Tubulin beta-5 chain [OS=Mus musculus] |
| P68372 | Tubb4b | Tubulin beta-4B chain [OS=Mus musculus] |
| P68369 | Tuba1a | Tubulin alpha-1A chain [OS=Mus musculus] |
| P68368 | Tuba4a | Tubulin alpha-4A chain [OS=Mus musculus] |
| Q62191 | Trim21 | E3 ubiquitin-protein ligase TRIM21 [OS=Mus musculus] |
| Q9JJZ2 | Tuba8 | Tubulin alpha-8 chain [OS=Mus musculus] |
| Q99JY9 | Actr3 | Actin-related protein 3 [OS=Mus musculus] |
| P54987 | Acod1 | Cis-aconitate decarboxylase [OS=Mus musculus] |
| Q8JZX4 | Rbm17 | Splicing factor 45 [OS=Mus musculus] |
| P19973 | Lsp1 | Lymphocyte-specific protein 1 [OS=Mus musculus] |
| P25911 | Lyn | Tyrosine-protein kinase Lyn [OS=Mus musculus] |
| P16951 | Atf2 | Cyclic AMP-dependent transcription factor ATF-2 [OS=Mus musculus] |
| Q9R112 | Sqor | Sulfide:quinone oxidoreductase, mitochondrial [OS=Mus musculus] |
| P60843 | Eif4a1 | Eukaryotic initiation factor 4A-I [OS=Mus musculus] |
| P97793 | Alk | ALK tyrosine kinase receptor [OS=Mus musculus] |
| P61161 | Actr2 | Actin-related protein 2 [OS=Mus musculus] |
| P16460 | Ass1 | Argininosuccinate synthase [OS=Mus musculus] |
| Q925I1 | Atad3 | ATPase family AAA domain-containing protein 3 [OS=Mus musculus] |
| P63017 | Hspa8 | Heat shock cognate 71 kDa protein [OS=Mus musculus] |
| Q64213 | Sf1 | Splicing factor 1 [OS=Mus musculus] |
| Q6NXH9 | Krt73 | Keratin, type II cytoskeletal 73 [OS=Mus musculus] |
| Q02257 | Jup | Junction plakoglobin [OS=Mus musculus] |
| Q91YQ5 | Rpn1 | Dolichyl-diphosphooligosaccharide--protein glycosyltransferase subunit 1 [OS=Mus musculus] |
| P38647 | Hspa9 | Stress-70 protein, mitochondrial [OS=Mus musculus] |
| Q8BMJ8 | Sp8 | Transcription factor Sp8 [OS=Mus musculus] |
| Q62167 | Ddx3x | ATP-dependent RNA helicase DDX3X [OS=Mus musculus] |

| Accession | Gene name | Description |
|---|---|---|
| Q9WUA3 | Pfkp | ATP-dependent 6-phosphofructokinase, platelet type [OS=Mus musculus] |
| A2BIM8 | Mup18 | major urinary protein 18 [OS=Mus musculus] |
| Q8K1L0 | Creb5 | Cyclic AMP-responsive element-binding protein 5 [OS=Mus musculus] |
| Q9WUM4 | Coro1c | coronin-1C [OS=Mus musculus] |
| Q61233 | Lcp1 | Plastin-2 [OS=Mus musculus] |
| P20029 | Hspa5 | 78 kDa glucose-regulated protein [OS=Mus musculus] |
| Q7TPR4 | Actn1 | Alpha-actinin-1 [OS=Mus musculus] |
| E9Q634 | Myo1e | Unconventional myosin-Ie [OS=Mus musculus] |
| Q9WU78 | Pdcd6ip | Isoform 3 of Programmed cell death 6-interacting protein [OS=Mus |
| Q6IFX2 | Krt42 | Keratin, type I cytoskeletal 42 [OS=Mus musculus] |
| O55143 | Atp2a2 | Sarcoplasmic/endoplasmic reticulum calcium ATPase 2 [OS=Mus musculus] |
| P11499 | Hsp90ab1 | Heat shock protein HSP 90-beta [OS=Mus musculus] |
| P97449 | Anpep | Aminopeptidase N [OS=Mus musculus] |
| Q9Z331 | Krt6b | Keratin, type II cytoskeletal 6B [OS=Mus musculus] |
| O35691 | Pnn | Pinin [OS=Mus musculus] |
| Q9JKF1 | Iqgap1 | Ras GTPase-activating-like protein IQGAP1 [OS=Mus musculus] |
| Q99104 | Myo5a | Unconventional myosin-Va [OS=Mus musculus] |
| Q6R891 | Ppp1r9b | Neurabin-2 [OS=Mus musculus] |
| Q8BTM8 | Flna | Filamin-A [OS=Mus musculus] |
| Q80SU7 | Gvin1 | Interferon-induced very large GTPase 1 [OS=Mus musculus] |
| Q0P678 | Zc3h18 | Zinc finger CCCH domain-containing protein 18 [OS=Mus musculus] |
| B2RQC6 | Cad | CAD protein [OS=Mus musculus] |
| Q8CH25 | Sltm | SAFB-like transcription modulator [OS=Mus musculus] |
| P62983 | Rps27a | Ubiquitin-40S ribosomal protein S27a [OS=Mus musculus] |

## Transparent Methods

### Animals and mouse model

The *Fcgr2b*- deficient mice on the 129/ C57BL/ 6 background (MGI Cat# 2448997, RRID: MGI: 2448997) were obtained from Dr. Bolland (NIH, Maryland, USA). Sting-deficient mice (MGI Cat# 4939598, RRID: MGI: 4939598) were provided by Paludan (Aarhus University, Aarhus, Denmark). Wild type (WT) mice were purchased from the National Laboratory Animal Center, Nakornpathom, Thailand. The *Fcgr2b*- deficient mice were crossed with *Sting*- deficient mice to generate the double-deficient mice and their littermate controls. The double-deficient mice were aged up to 12 months and observed survival compared to their littermates. The animal protocols were approved by the Animal Experimentation Ethics Committee of Chulalongkorn University Medical School.

### Measurement of autoantibody

Blood was collected from the mice at the age of 6- 7 months. The levels of anti-dsDNA

from sera (dilution 1:100) were measured by Enzyme-linked immunosorbent assay (ELISA). The anti-nuclear antibodies in serum (dilution 1:800) were detected by indirect immunofluorescence using HEp- 2 cells (EUROIMMUN, Luebeck, Germany). Samples were showed fluorescence intensity and blindly graded as 4= maximal fluorescence (brilliant yellow-green), 3 = less brilliant (yellow- green fluorescence), 2= definite (dull yellow- green), and 1= very dim (subdued fluorescence).

**Measurement of cGAMP production**

Total splenocytes were isolated from WT, *Sting*-deficient, *Fcgr2b*-deficient, and double deficient mice. Spleens were dispersed through a cell strainer to generate a single-cell suspension and eliminated red blood cells by osmotic shock (ACK buffer: $NH_4Cl$, $KHCO_3$, and EDTA). The splenocytes were lysed in M-PER™ extraction buffer (Thermo Fisher Scientific, MA USA) (20 x $10^6$ cells/100 ul of lysis buffer). cGAMP in cells lysate was measured using 2'3'-cGAMP ELISA Kit (Cayman Chemical, Michigan, USA) according to the manufacturer's instructions. In brief, the ELISA plate contain a blanks (Blk), Total Activity (TA), non-specific binding wells (NSB), maximum binding wells (B0), an eight-point of standard curve and samples (cells lysates). Then, add 50 ul of 2'3'-cGAMP-HRP Tracer to each well except the Total Activity (TA) and the Blank (Blk) wells and add 50 ul of 2'3'-cGAMP Polyclonal Antiserum to each well except the TA, NSB, and the Blk wells. Then cover the plate with plastic film and incubate overnight at 4°C. After incubation, the plates were washed five times with 1x wash buffer, dried, and added 175 µl of TMB substrate solution, then incubated at room temperature for 30 minutes. The stop solution was then added (75 ul), and the absorbance was measured by Varioskan Flash Microplate Reader at 450 nm. (Thermo Fisher Scientific, MA USA).

**Cytokines detection**

Cytokine panels in serum including IL- 1α, IL- 1β, IL- 6, IL-10, IL-12p70, IL-17A, IL-23, IL-27, MCP- 1, IFN- β, IFN- γ, TNF- α, and GM- CSF were measured using LEGENDplex™ Mouse Inflammation Panel kit ( Biolegend, San Diego, CA, USA) according to the manufacturer's instructions. The beads were read on a flow cytometer using BDTM LSR- II (BD Biosciences, USA) and analyzed by LEGENDplexTM Data Analysis Software.

**Single-cell preparations**

Splenic cells were isolated from all experimental groups and littermate WT mice. Spleens were dispersed through a cell strainer to generate a single-cell suspension and eliminated red blood cells by osmotic shock (ACK buffer: $NH_4Cl$, $KHCO_3$, and EDTA). $CD4^+$ T cells and naïve T cells were isolated from spleen using CD4 isolation Kit and naïve $CD4^+$ T cell Isolation Kit (Miltenyi, Bergisch Gladbach, Germany) as per manufacturer's instructions.

**Flow cytometry analysis**

The splenocytes ($1 \times 10^6$ cells) were stained with flow antibody including anti-CD4 (clone: GK1. 5; cat.100423), CD8 (clone: 53-6. 7; cat. 100708), CD62L (clone: MEL-14; cat. 104417), CD44 (clone: IM7; cat.103035), CD3ε (clone: 145-2C11; cat.100312), ICOS ( clone: C398.4A; cat. 313517), CD11c (clone: N418; cat. 117312), B220 (clone: RA3-6B2; cat. 103222), CD11b (clone: M1/70; cat. 101228), I-Ab (clone: AF6-120.1; cat. 116406), PDCA-1 (clone: 129c1; cat.127103), CD80 (clone: 16-10A1; cat. 104733), GL7 (clone: GL7; cat. 144604), CD138 (clone:281-2; cat. 142506) (Biolegend, San Diego, CA, USA). The flow cytometry was performed using BD™ LSR-II (BD Biosciences, USA) and analysis by FlowJo software (USA).

Intracellular staining of T cell was performed as the following method. $CD4^+$ cells ($2 \times 10^5$ cells) were plated in 200 μl of complete medium supplemented with 25 ng/ml PMA, 1 μg/ml ionomycin (Sigma-Aldrich, Darmstadt, Germany) and 1X GolgiPlug (brefeldin A, Biolegend). After 4 hours of incubation at $37^0$C and 5% $CO_2$, cells were collected and stained with anti-CD3, anti-CD4, anti-CD28, and ICOS before following the cytokines intracellular staining. Briefly, after performed cell surface antigen staining, cells were fixed in 200 μl of fixation buffer (Biolegend) in the dark at $4^0$C overnight. Cells were washed three times with 1X permeabilization buffer (Biolegend) and resuspend fixed/permeabilized cells in 50 μl of 1X permeabilization buffer, then added the fluorophore-conjugated antibody for anti- IFN- γ ( clone: XMG1. 2: cat. 505821) (Biolegend, San

Diego, CA, USA). The flow cytometry was performed using BD$^{TM}$ LSR- II (BD Biosciences, USA) and analysis by FlowJo software (USA).

### Gene expression analysis

A total of RNA was extracted from the kidneys and spleen by Trizol reagent (Invitrogen, CA, USA) as per manufacturer's instructions followed by RNA purified using the RNeasy mini kit and treated with DNase I (Qiagen, MD, USA). Then, 1 μg of total RNA was used as a template for cDNA synthesis using iScript RT Supermix (Biorad, California, USA). The expression of interest genes was assessed by quantitative real-time PCR. The gene expression profiles were tested using SsoAdvanced Universal SYBR Green Supermix (Biorad, California, USA). The thermal cycling conditions are as follows: 1 cycle of 95 0C for 5 minutes, followed by 40 cycles of 95 0C for 15 seconds and 60 0C for 1 minute. The relative amounts of target mRNA will be normalized to β-actin mRNA as a housekeeping gene and determined by the $2^{(-ddCt)}$. Microarray analysis was performed by RNA labeling and hybridization using the Agilent One-Color Microarray- Based Gene Expression Analysis protocol (Agilent Technology, V 6. 5, 2010). Microarray results were extracted using Agilent Feature Extraction software v11. 0 (Agilent Technologies, Palo Alto, USA).

### Immunofluorescence and Histopathology

Frozen renal sections were fixed in acetone and blocked with 1% BSA in PBS. After that, the sections were stained with FITC-conjugated goat anti- mouse IgG antibodies and PE-conjugated goat anti-mouse C45RB antibodies (Abcam, Cambridge, MA, USA). Then, samples were stained with DAPI (4',6- Diamidino- 2- Phenylindole, Dihydrochloride) (Thermo Fisher Scientific, MA USA) for 5 minutes in the dark at room temperature. Slides were washed three times and mounted with ProLongTM diamond antifade mountant (Invitrogen, CA, USA). The fluorescent signaling was visualized by ZEISS LSM 800 with Airyscan (Carl Zeiss, Germany).

For determine the $CD45^+$ cell deposition and C3 deposition in the kidney, the sections were stained with anti-C3c antibody (FITC; cat. Ab4212) (Abcam, Cambridge, MA, USA), Alexa Fluor® 647 anti-mouse CD3 (clone: 17A2; cat. 100209) Antibody and Alexa Fluor® 647 anti-mouse CD11c (clone: N418; cat. 117312) (Biolegend, San Diego, CA, USA). Then, samples were stained with DAPI, as described above. The quantification of fluorescence intensity was analyzed by ZEISS ZEN Microscope Software (Carl Zeiss, Germany). The deparaffinized kidney sections were fixed with formalin subsequently stained with H&E. The pathology grading from kidney sections was blinded analysis by the nephrologist.

**Cell preparation for in vitro assay**

Differentiation of bone marrow-derived dendritic cell (BMDC) was performed as follows. Bone marrow cells were obtained from the femur. Cells were cultured in RPMI 1640 supplemented with 10 % FBS, 1 mM Na pyruvate, 10 mM HEPES buffer, 1% L-glutamine, 1% nonessential amino acid, 100 units/ml pen/ strep (Gibco-Thermo Fisher Scientific, MA USA) and 50 μM 2-mercaptoethanol (Sigma-Aldrich, Darmstadt, Germany). Cells were stimulated with IL-4 (20 ng/ml) and GM-CSF (20 ng/ml) (Miltenyi, Bergisch Gladbach, Germany) and maintained at 370C in a CO2 incubator for five days. The immature dendritic cells were activated by adding 10 μg/ml of DMXAA (5,6-Dimethylxanthenone-4-acetic acid or STING ligand) (Invivogen, San Diego, USA) for 24 hours. The mature BMDC and collected supernatant were stained with the antibodies and analyzed by flow cytometer. For cGAMP activation,BMDC from WT, $Sting^{gt/gt}$, $Fcgr2b^{-/-}$ and $Fcgr2b^{-/-}$. $Sting^{gt/gt}$ were cultured, as described above. The immature dendritic cells were transfected with 4 μg/ml of 2'3'-cGAMP (cyclic GMP-AMP; c-GpAp) (Invivogen, San Diego, USA) by lipofectamine P3000 Reagent (Thermo Fisher Scientific, MA USA) as per manufacturer's instructions for 24 hours. The mature BMDC were stained with the antibodies and analyzed by flow cytometer.

CFSE (Carboxyfluorescein succinimidyl ester) labeling was performed as described below. T cells (1x $10^6$) in pre-warmed (37$^0$C) in PBS were incubated with 0. 5 μM CFSE (Biolegend, San Diego, CA, USA) at 37$^0$C in a $CO_2$ incubator for 10 minutes. Cells were quench labeling reaction with ten volumes of complete ice-cold medium and centrifuged for 5 minutes at 1,500 rpm, 4$^0$C. Labeled cells were washed by resuspending in a complete medium. CFSE labeled cells are now ready for in vitro culture.

**In vitro co-cultures of BMDCs and T cells**

BMDCs were cultured with T cells for 6 hours. Briefly, immature BMDCs were cultured and activated with DMXAA (STING ligand) for 24 hours at 37$^0$C and washed twice before co-culture. Activated BMDCs (4 x $10^4$) were plated in 200 μl of complete medium with CD4+ T cells (2 x $10^5$) from lymph nodes (1:5; 4 x$10^4$ DC: 2 x $10^5$ T cells) for 6 hr at 37$^0$C and 5% $CO_2$ followed by intracellular staining for anti-IFN-γ as described above. For proliferation assays, naïve T cells were labeled with CFSE as described above, according to the manufacturer's instruction (Biolegend, San Diego, CA, USA), before co-cultured with activated BMDCs. In brief, activated BMDCs (4 x $10^4$) were plated in 200 μl of complete medium with naïve T cells (2 x $10^5$) from spleen (1:5, 4 x$10^4$ DC: 2 x $10^5$ T cells) for 72 hours at 37$^0$C and 5% $CO_2$. After co-culture, T cells were stained with intracellular cytokine for anti-IFN-γ and detect their proliferation by dilution of CFSE fluorescence by flow cytometer.

**Colocalization of STING and LYN in BMDC cells**

Immature BMDCs from WT and *Fcgr2b*$^{-/-}$ were cultured, then stimulated with DMXAA for 6 hr. Cells were fixed with 4% formalin (Sigma-Aldrich, Darmstadt, Germany) at room temperature for 15 minutes. Incubate the fixed cells for 10 min with 0.2% Triton X-100 and block unspecific binding of the antibodies with 0.1% BSA for 1 hour BMDCs were probed with anti-LYN antibody (cat. 2732, Cell Signaling, MA, USA) and anti-FYN antibody [FYN-01] (cat. 1881, Abcam, Cambridge,

MA, USA) for overnight at 4 $^{0}$C. After incubation, secondary antibody Alexa Fluor 488 rabbit IgG (Thermo Fisher Scientific, MA, USA) was added for 1 hour at room temperature. For STING detection, STING antibody was incubated with Zenon$^{TM}$ Alexa fluor$_{TM}$ 555 rabbit IgG labeling kit (Thermo Fisher Scientific, MA, USA) and then stained the BMDCs. The cells were subsequently probed with 1 μM DAPI (Thermo Fisher Scientific, MA USA) for 5 minutes, and the fluorescent signaling was visualized by ZEISS LSM 800 with Airyscan (Carl Zeiss, Germany). To confirm the specificity of the antibody, BMDCs were probed with LYN antibody (clone Lyn-01; cat MAB0949) (1:200) (Abnova,Taipei, Taiwan) and STING-C antibody (clone D2P2F, cat: 13647) (Cell Signaling, MA, USA) as described above. The colocalization score was analyzed by ZEISS ZEN Microscope Software (Carl Zeiss, Germany).

**Sample preparation for MS analysis**

Quantitative proteomic analysis of mature BMDC was studied using a dimethyl labeling method (Makjaroen et al., 2018). Briefly, BMDC were cultured and then stimulated with 10 µg/ml of DMXAA (STING ligand) for 24 hours. Three hundred microgram proteins per group from BMDC were digested overnight at 37 $^{0}$C with trypsin [1:50 (w/w)]. Next, stimulated BMDC's peptides from WT mice, *Fcgr2b*$^{-/-}$ mice, and *Fcgr2b*$^{-/-}$.*Sting*$^{gt/gt}$ mice were labeled with light reagents [formaldehyde (Sigma) and sodium cyanoborohydride (Sigma)], medium reagents [formaldehyde-d2 (CIL) and sodium cyanoborohydride], and heavy reagents [deuterated and 13C-labeled formaldehyde (Sigma) and cyanoborodeuteride (CIL)], respectively, for an hour at room temperature. The peptides were fractionated and subjected to LC-MS/MS (Thermo). Unpaired t-tests determined significantly differentially regulated proteins (with p-value < 0.05 considered significant).

For proteomics analysis, the WT mice channels were used as denominators to generate abundance ratios of *Fcgr2b*$^{-/-}$ mice/ WT mice and *Fcgr2b*$^{-/-}$.*Sting*$^{gt/gt}$ mice/ WT mice**.** Log2 of the

normalized ratio was used to calculate the mean and standard deviation of fold change across all four biological replicates. When these ratios were found in less than three experiments, the relevant proteins were excluded. To analysis the up- and down-regulated proteins, the ratios of *Fcgr2b*$^{-/-}$/*Fcgr2b*$^{-/-}$.*Sting*$^{gt/gt}$ mice were performed. Unpaired t-tests determined significantly differentially regulated proteins with p-value < 0.05 considered significant. The online resource Database for Annotation, Visualization, and Integrated Discovery (DAVID, v 6.8, https://david.ncifcrf.gov/), and interferome (http://www.interferome.org/) was employed to classify the vital proteins into functional categories and interferon regulated proteins using all proteins identified by MS as background (for DAVID).

**Isolation of T cells from the lymph node and spleen**

Lymph nodes and spleens were removed from sacrificed mice and littermate wild-type mice. Lymph nodes and spleens were dispersed through a cell strainer to generate a single-cell suspension as described above. T cells were isolated and purified using CD4$^{+}$ T Cells Isolation Kit (Miltenyi, Bergisch Gladbach, Germany) as per manufacturer's instructions. Briefly, splenocytes and total cells from lymph nodes were prepared (2 x 107) and incubated with 20 μl of Biotin-Antibody Cocktail for 5 minutes at 4 $^{0}$C. Then, the mixed cells were incubated with 40 μl of Anti- Biotin MicroBeads for 10 minutes at 4 $^{0}$C, and proceed to subsequent magnetic cell separation (Miltenyi, Bergisch Gladbach, Germany). The viability and purity of purified CD4+ T cells were stained with anti-CD3, anti-CD4 (Biolegend, San Diego, CA, USA) and Fixable Viability Dye eFluor® 780 (Thermo Fisher Scientific, MA USA), then examined by flow cytometer.

**Immunoprecipitation of STING-interacting proteins**

BMDCs from WT, *Fcgr2b*$^{-/-}$ and *Sting*$^{gt/gt}$ were cultured, then stimulated with DMXAA for 3 hr. Cells were collected and lysed with 1 % IGEPAL CA-630, 0.5% TritonX-100, 150 mM NaCl, 50 mM Tris pH 7.4, 5% glycerol, 100 mM beta-Glycerophosphate, 2 mM $Na_3VO_4$ and 1X proteases

inhibitor cocktail (Roche). First, the antibody was mixed with the magnetic beads by adding 10 µg of STING antibody (CUSB in-house antibody, clone: GTN-01; targeted N-terminal) with 400 µg of SureBeads™ Protein A magnetic beads (Biorad, California, USA) and incubated for 1 hour at room temperature. Then, Protein lysates were added and incubated with antibody-conjugated beads for overnight at 4$^0$C. After incubation, the beads were washed three times with wash buffer (150 mM NaCl and 50 mM Tris- HCl pH 7. 4). Samples were eluted by adding 5X laemmli buffer and boiled 95$^0$C for 10 minutes. For reverse IP, the antibody was mixed with the magnetic beads by adding 10 µg of LYN antibody (clone Lyn-01; cat MAB0949) (Abnova, Taipei, Taiwan) with 400 µg of SureBeads™ Protein G magnetic beads (Biorad, California, USA) followed by incubated with activated BMDC as described above. The eluted protein samples were separated by 10 % SDS- PAGE gel. The STING-interacting proteins from co- IP were analyzed by in-gel digestion, followed by LC-MS/MS analysis.

**Western Blot Analysis**

Splenocytes were lysed in 2 % SDS lysis buffer. Lysates were homogenized and centrifuged at 12,000×g for 15 min at 4 $^0$C. The supernatants were collected, and total protein was measured by BCA protein assay (Thermo Scientific, Illinois, USA). Cell lysates containing equal amounts of protein (20 µg) were boiled in SDS sample buffer at 37 $^0$C for 15 min before separation on a 10 % SDS-polyacrylamide gel. Proteins were transferred to nitrocellulose membranes and Western blot analysis.

BMDC from WT, *Fcgr2b*$^{-/-}$, and *Sting*$^{gt/gt}$ was cultured and stimulated with DMXAA for 3 hours, as described above. Protein lysates were prepared and run on 10 % SDS-polyacrylamide gel, then proteins were transferred to nitrocellulose membranes and probed with STING antibody (clone: GTN-01; 1:2000) (CUSB-in house, BKK, TH), STING-C antibody (clone D2P2F, cat: 13647) (Cell Signaling, MA, USA), LYN antibody (clone: LYN-01;1:1000) (Biolegend, San Diego, CA, USA) and LYN antibody (cat: 2732) (Cell Signaling, MA, USA). After incubation at 4 $^0$C for overnight,

the membrane was washed and probed with IRDye® 680RD Donkey anti-Rabbit IgG (H + L) and IRDye® 800CW Donkey anti-Mouse IgG (H + L) secondary antibody (1:10000) ( LI-COR, Lincoln, Nebraska, USA) for 1 hour at room temperature. The membrane was determined the signals by ODYSSEY CLx (LI-COR, Lincoln, Nebraska, USA).

**Inhibition of LYN in Sting-activated BMDC**

BMDC was prepared as described above in the presence of GM-CSF and IL-4 (Miltenyi, Bergisch Gladbach, Germany) in a 100 mm petri dish (at least 5 million cells/dish). On the day of DMXAA stimulation, free and non-adherent cells were flushed off and spun down, counted, and seeded out into 24-well plates (approx. 1 mill cells/well). Cells were then incubated with the LYN inhibitors PP2 (Sigma-Aldrich, Darmstadt, Germany) for one hour before the addition of DMXAA and LPS for 24 hrs, and subsequently stained and fixed for flow cytometry analysis.

For protein expression, total proteins from BMDC were analyzed by Western blotting. Samples were diluted in XT sample buffer and XT reducing agent and ran on an SDS–PAGE (Criterion™ TGX™). Trans-Blot Turbo™ Transfer System® was used for the transfer of proteins to PVDF membranes (all reagents Bio-Rad). The membrane was blocked in 5% Difco™ skim milk (BD). The antibodies used for Western blotting were all from Cell Signaling (Cell Signaling, MA, USA), Phospho-LYN (Tyr507) Rabbit Antibody, CST-2796S, LYN (C13F9) Rabbit mAb, CST-9271S, Phospho-AKT (Ser473) Antibody, and CST-AKT antibody. Quantification of the western blot was analyzed by ImageJ software. For gene expression, a total RNA from BMDC after DMXAA stimulation with or without PP2 inhibitor for 3 hours was isolated by Trizol reagent (Invitrogen, CA, USA) as described above. The interferon signature genes were tested by real-time PCR.

## Adoptive transfer

BMDC from WT, *Fcgr2b*$^{-/-}$, and *Fcgr2b*$^{-/-}$.*Sting*$^{gt/gt}$ mice were cultured as described above. The recipient WT or *Fcgr2b*$^{-/-}$.*Sting*$^{gt/gt}$ mice (at the age of 4 months) received approximately $10^6$ cells of BMDC via tail vein injection every two weeks per injection for four times. The control, *Fcgr2b*$^{-/-}$.*Sting*$^{gt/gt}$ mice received only sterile PBS (vehicle). Sera were collected, and ELISA measured the levels of anti-dsDNA. Mice were euthanized two weeks after the final transfer (at the age of 6 months).

## Statistical analysis

All statistical analyses employed the two-tailed Mann-Whitney test. Statistical analyses were performed using GraphPad Prism 4.0 (GraphPad Software, San Diego, CA).

## Supplemental information

Gene expression profiles of the kidneys have been deposited in the Gene expression Omnibus (GEO) website with ID GSE142594 and available at https://www.ncbi.nlm.nih.gov/geo/query/acc.cgi?acc=GSE142594

The mass spectrometry proteomics data, including annotated spectra for all modified peptides and proteins identified, have been deposited to the ProteomeXchange Consortium via the PRoteomics IDEntifications (PRIDE) partner repository with the data set identifier PXD019239.

## Supplemental References

Makjaroen, J., Somparn, P., Hodge, K., Poomipak, W., Hirankarn, N., and Pisitkun, T. (2018). Comprehensive Proteomics Identification of IFN-lambda3-regulated Antiviral Proteins in HBV-transfected Cells. Mol Cell Proteomics *17*, 2197-2215.
